# Biodiversity of Indigenous *Saccharomyces* Populations from Old Wineries of South-Eastern Sicily (Italy): Preservation and Economic Potential

**DOI:** 10.1371/journal.pone.0030428

**Published:** 2012-02-29

**Authors:** Sabina Di Maio, Giuseppe Polizzotto, Enrico Di Gangi, Giusy Foresta, Giuseppe Genna, Antonella Verzera, Antonio Scacco, Gabriele Amore, Daniele Oliva

**Affiliations:** 1 Istituto Regionale della Vite e del Vino, Palermo, Italy; 2 Dipartimento di Chimica Organica e Biologica, Università di Messina, Messina, Italy; 3 Dipartimento di Scienze Agrarie e Alimentari, Università di Catania, Catania, Italy; 4 Animal Physiology and Evolution Laboratory, Stazione Zoologica “Anton Dohrn” Napoli, Napoli, Italy; Universidade de Sao Paulo, Brazil

## Abstract

In recent years, the preservation of biodiversity has become an important issue. Despite much public discussion, however, current practices in the food industry seldom take account of its potential economic importance: on the contrary, the introduction of industrialized agriculture practices over large areas has often resulted in a dramatic reduction in biodiversity.

In this paper, we report on the remarkable degree of biodiversity in the wine yeast populations naturally present in a small area of Sicily (Italy) where traditional (non-industrial) winery practices are still in place. Out of more than 900 *Saccharomyces* yeast isolates recovered from late spontaneous fermentations, we detected at least 209 strains. Most interestingly, when evaluated at the fermentation and technological level, a number of isolates were found to be superior to industrial yeast strains. Out of a selected group, isolates from two strains were used for experimental fermentations in a winery environment and the quality of the wines produced was assessed at the technological, quality and sensory levels. Given that the characteristics of the wines produced were found to be industrially appealing, the study demonstrated the economic potential of preserving the patrimony of Sicilian yeast biodiversity and highlighted the importance of maintaining traditional wine making practices.

## Introduction

Since the introduction of the term “biodiversity” there has been intense, public discussion regarding the value and benefits of its preservation. On the practical side, however, biodiversity conservation has often clashed with the economic interests of the food industry, as dedicating large tracts of land to single cultures for the purposes of mass food production has the ineluctable consequence of reducing biodiversity. This has become one of the most important arguments against GMOs, and the large-scale utilization of standardized seed is perceived as a major threat to the preservation of crop biodiversity in developing countries. However it is not always clear how the food industry would benefit from preserving biodiversity.

In this study, we have focused on a small area in south-eastern Sicily where traditional (“spontaneous”) wine fermentation practices are still in place and industrial yeast strains have not yet replaced native ones. We report on the remarkable degree of biodiversity in yeast populations present in the area and indicate how this can potentially benefit the local wine industry.

During spontaneous fermentation, populations of different yeast genera live together and succeed one another. The early stages of fermentation are usually dominated by *Hanseniaspora*/*Kloeckera* and often show the presence of *Candida*, *Metschnikowia*, *Pichia*, *Rhodotorula* and *Torulaspora*, while alcoholic fermentation is completed by strains of the *Saccharomyces* genus [1], [2].

In an attempt to improve and standardize the characteristics of wines, industrially produced strains of *Saccharomyces* are used in many wineries. However, the introduction of these strains can have a great impact on the diversity of local yeast populations [3], [4], with the loss of a patrimony of yeast biodiversity once typical of areas with a history of wine-making going back hundreds or even thousands of years. Studying the diversity of yeasts can shed light on their population dynamics [5], [6], lead to the discovery of strains with novel enological characteristics of industrial value [7]–[9] and help produce wines typical of specific areas.

We studied *Saccharomyces* populations responsible for wine fermentation in six different wineries in south-eastern Sicily, an area with a history of wine production dating back more than two thousand years [10], [11]. We recovered more than 900 yeast isolates from the musts fermented inside the traditional stone-concrete troughs still in use in these wineries. We characterized our isolates by molecular techniques such as restriction fragment length polymorphism analysis of mitochondrial DNA (mt-DNA RFLP [12]) and amplification of specific chromosomal DNA sequences (δ-PCR [13]). On the basis of the different mt-DNA RFLP band patterns obtained, we were able to identify at least 209 strains.

To highlight the value of such diversity, we analyzed some of the “desirable characteristics of wine yeast” [1] of the isolates belonging to 129 of these strains. We measured the capacity for prompt and rapid fermentation (fermentation vigor), fermentation vigor maintenance in the presence of sulfur dioxide (sulfite tolerance) and ethanol production (fermentation power). We also assessed growth patterns, and the ability to sporulate, which is important both for taxonomic and commercial purposes, as it facilitates the hybridization between different strains [1], [14]. Furthermore we considered quality features such as low sulfide and volatile acid production [15], and the expression of specific enzymatic activities such as β–glucosidase, which liberates terpenols from their terpenyl-glycoside precursors and intensifies the varietal character of a wine [16]. In the vast majority of cases, the strains we recovered possessed technological and quality characteristics comparable (or even superior in a number of cases) to those of commercially available yeast strains. Two selected strains were then used as starters for 100-liter fermentations on “Nero d'Avola” and “Frappato” musts. Thus the results of the study illustrate how preserving yeast biodiversity can preserve a biological patrimony of great interest, not only in terms of basic research but also with regard to possible industrial applications.

## Materials and Methods

### Yeast strains

The *S. cerevisiae* strain L404 and 6167 and the *S. bayanus* strain 11719 belong to the DIPROVAL collection of the University of Bologna (commercialized by Oliver-Ogar, Italy). The *S. cerevisiae* EC1118, ICV D254, QD145 and RC212 strains are commercialized by Lallemand; the *S. cerevisiae* strains Zymaflore F10 and Zymaflore F15, are commercialized by Laffort; the *S. cerevisiae* Fermol Davis 522 and Fermol Arome Plus, by Pascal Biotec. The *S. cerevisiae* NDA21 strain is commercialized by Biospringer, Maison Alfort-France. The *Hanseniaspora uvarum* 1-03 strain belongs to the IRVV collection [17].

### Sampling

**Figure 1:**
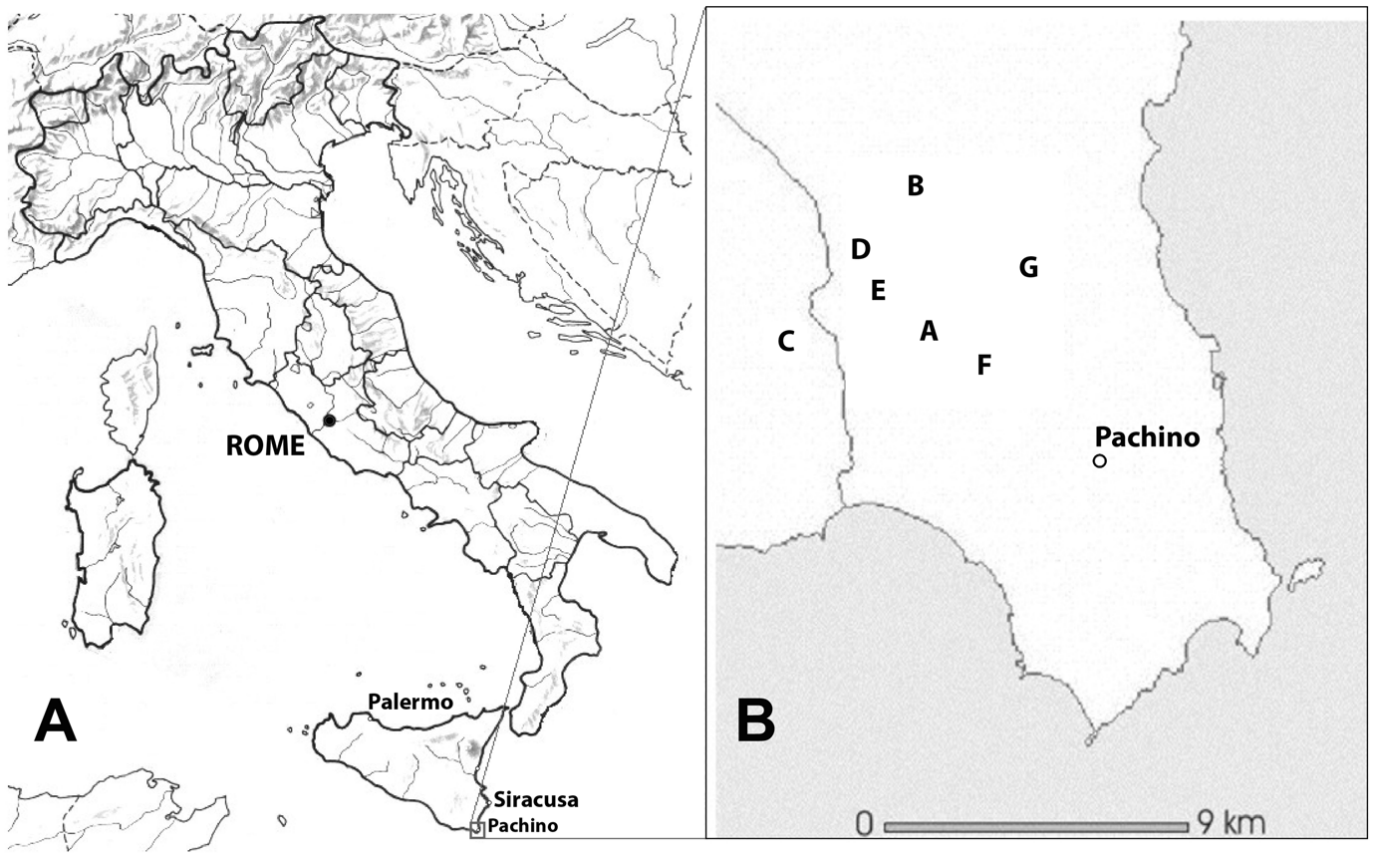
Research area (A) and location of the wineries (B) where must sampling was carried out (collection sites are indicated by capital letters).

Sample sites were chosen during the 2002 and 2003 harvests, on the basis of information provided by the technical personnel of each winery, ensuring that the musts included in the study came from wineries where commercial yeast strains were never used. Sampling area and sites (A–G) are shown in figure 1. From the 2002 harvest, four samples from site A (A1–A4), two samples from site B (B2 and B3) and one sample from site D (D1) were selected. From the 2003 harvest, eleven samples were obtained: three from site A (A5 to A7), three from site B (B4 to B6), two from site D (D2 and D3) and one from each of sites E, F and G (E1, F1 and G1).

Musts samples from stone-concrete fermentation troughs were put in sterile containers, a 50% (v/v) must∶glycerol mixture was obtained and rapidly stored at −80°C (for no longer than 8 months) to preserve microorganism viability.

*Saccharomyces* colonies were isolated as follows. Musts were sequentially diluted from 1∶10 to 1∶100,000 in 0.1% (w/v) sterile peptone. 0.2 ml of each dilution was spread on WL Nutrient Agar Oxoid. After four days in culture at 28°C, three colony morphologies were detected: 1-colonies with a creamy to greenish color and with a knob-like, opaque, smooth surface, typical of the *Saccharomyces/Torulaspora* genera [18]; 2-flat colonies of intense green color, smooth and opaque surface, typical of *Hanseniaspora/Kloeckera* genera [18]; 3-colonies with a dark intense green center, clear rim and domed surface, referred as *Candida stellata* [19] (and most probably belonging to the *Candida zemplinina* species [20]). Must samples with morphology 1 in a ratio of 20∶1 to the others, were selected for further analysis. At least 50 isolates were recovered from each fermentation batch: this represents a sufficient number for statistically significant analyses [12].

A total of 930 different colonies were numbered (from A1-1 to G1–52) and plated on Lysine Agar Oxoid. Of these, 918 isolates (352 from 2002 and 566 from 2003) were unable to utilize lysine as a nitrogen source and were therefore identified as representatives of the *Saccharomyces* genus (according to [21], [22]). The *S. cerevisiae* strain 6167 and the *H. uvarum* 1-03 strain were used as controls.. *S. cerevisiae* and *S. bayanus* are the most representative species found in late fermentation musts [1]; therefore the 918 *Saccharomyces* isolates were plated on vitamin-free media (Biolife-Italy), to identify *S. bayanus* yeasts (which grow on this medium; [22]). The *S. bayanus* 11719 and the *S. cerevisiae* 6167 strains were used as controls. No *S. bayanus* isolate was found. Therefore we provisionally assigned our 918 isolates to the *S. cerevisiae* species.

### Molecular analyses

Mt-DNA RFLP analyses were performed, essentially as described by [12] with some minor modification. After 70% (v/v) ethanol washing, the mt-DNA pellets were dissolved in 16 µl of distilled water. 8 µl of this solution were digested with 2.5 U of *RsaI* or *HinfI* restriction endonucleases (Biolabs). After adding 1 µl of 1 mg/ml RNase (Fluka) and a further incubation of 30 minutes at 37°C, samples in 1× TAE buffer were loaded onto a 0.7% (w/v) agarose gel. Gel images were acquired using a Gel Doc 2000 BioRad apparatus. Diversity Database™ software [23] was used to compare and distinguish between different mtDNA RFLPs patterns.

MtDNA RFLP analyses on lees were performed to ascertain that the *Saccharomyces* strains present in the musts at the end of fermentation were identical to those that were inoculated. These analyses were coupled with microbiological controls which confirmed that the vast majority of the yeasts proliferating in the musts were *Saccharomyces* (described in “Setup and analysis of experimental fermentations”). 100 µl of lees were diluted in 1 ml of YPD (10 g/l yeast extract, 20 g/l peptone, 20 g/l glucose, 30 ppm tetracycline) and grown at 28°C for 24–48 h. The mt-DNA of the yeast cells was digested, loaded on gel and the band pattern was compared with that of the inoculated starters (it is reported that this technique allows an accuracy of at least 90% based on the cleanness of the patterns obtained [24]).

The amplification of the δ interspersed sequences was performed using primers δ1 (5′-CAAAATTCACCTAT[A/T]TCTCA-3′) and δ2 (5′- GTGGATTTTTATTCCAACA-3′) [13]. The amplification reaction was carried out using 24 ng of template DNA, 0.2 mM dNTPs, 40 pmol of each primer, 1 U of Taq DNA polymerase (Promega) in the buffer supplied by the manufacturer with the addition of 2 mM MgCl_2_, in a final volume of 40 µl. Thermal cycling parameters were as in [25]. The size of the amplicons was estimated by electrophoresis in 1.4% (w/v) agarose gels, in 1× TAE buffer, using the 100 bp DNA ladder (BDH). DNA from the EC1118 yeast strain was used as positive control and a DNA-free sample as negative control. To avoid artifacts, faint non-reproducible bands were not considered for analysis [26].

To confirm that the B2–25 and B2–48 isolates belonged to the *S. cerevisiae* species, the ITS region was amplified using the ITS1 (5′–TCCCCCCGTAGGTGAACCTGCGG-3′) and ITS4 (5′-TCCTCCGCTTATTGATATGC-3′) primers [27]. An aliquot of the reaction was digested with 3 U of the *Hae*III restriction endonuclease. Upon digestion, all the amplicons produced four fragments of 320, 225, 180 e 145 bps, typical of the *S. cerevisiae* and *S. paradoxus* species. A *S. cerevisiae*-specific PCR reaction was then performed with the SC1 (5′-AACGGTGAGAGATTTCTGTGC-3′) and SC2 (5′-AGCTGGCAGTATTCCCACAG-3′) primers, as described in [28].

### Phenotypic characterization

Fermentation vigor and sulfite tolerance were assessed according to [14]. The L404 strain was used as positive control and non-inoculated bottles as negative control. Fermentation vigor was measured as weight loss due to CO_2_ production (gCO_2_/100 ml) after two and seven days of incubation at 25°C and expressed as relative values compared with L404. Sulfite tolerance was measured as fermentation vigor upon potassium metabisulfite supplementation (200 mg/l). To obtain an indication of fermentation power, tests were performed by supplementing musts with glucose up to a final concentration of 300 g/l of sugars [14]. Weight loss (due to CO_2_ production) was measured every day until the daily decrease was lower than 0.01 g. For each measurement standard errors were below 1%.

Growth patterns (defined according to [1]) were evaluated by visual inspection of samples using a Zeiss Axioscope2-Plus microscope.

To measure foam production, 100 ml cultures were grown in bottles and then agitated by hand.

To test for sporulation, isolates from each different strain were grown at 30°C for 7 days on acetate agar (5 g/l sodium acetate; 20 g/l agar) as described in [14]. Microscopic specimens were stained for 30 s in 5% (w/v) malachite green, washed and stained for 30 s in 0.5% (w/v) safranine [29]. Blue-colored spores and red-colored vegetative cells were distinguished using a Zeiss Axioscope2-Plus microscope.

Strains characterized by the production of high levels of acetic acid were identified by the halo produced on calcium carbonate agar plates after 7 days incubation at 25°C [14]. The L404 was used as a negative control and the *Hanseniaspora uvarum* 1-03 strain as a positive control.

Sulfide production was evaluated by color assessment of the yeast biomass (white, pale hazel, hazel, dark hazel, black) after a 2 days incubation at 25°C on BiGGY Agar as described in [15].

β-glucosidase production was assayed as in [16] by observing the browning of the yeast biomass after a 5 days incubation at 30°C on a medium containing 6.7 g/l Yeast Nitrogen Base (Difco), 5 g/l arbutin (Sigma), 0.2 g/L ammonium ferric citrate and 20 g/l agar (pH 5.0).

### Setup and analysis of experimental fermentations

Experimental fermentations (100 liters) were performed during the harvests of 2004 and 2006. All grapes came from Sicilian locations: for the 2004 fermentations “Nero d'Avola” grapes were obtained from Roano-Monreale (PA, Italy); for the 2006 fermentations, “Nero d'Avola” grapes were collected from Ceuso-Salemi (TP, Italy); “Frappato” grapes were obtained from Puntaloro-Ispica (RG, Italy). Grapes were delivered to the IRVV's Experimental Winery of Marsala (TP, Italy) and were de-stemmed and crushed. For these experiments, a total of 23 fermentations were performed: 15 fermentations in 2004 (14 strains were tested and compared with the control strain F10); 8 fermentations in 2006 (the B2–25 and B2–48 strains were used as starters on Nero d'Avola and Frappato musts and compared with the commercial strains ICV-D254 and QD145).

Potassium metabisulfite (0.1 g/l) was added to the musts and oenochemical analyses were performed. Pure yeast cultures were obtained by pre-multiplication in sterile must (11.1 °Baumé, pH 3.20) obtained by dilution of concentrated must. Musts were homogenized. Aliquots of 100 l for each cultivar were taken and inoculated with a liquid culture of each of the different isolates (at 5% v/v). Crushed grapes were allowed to ferment at 25°C. Throughout fermentations, the amount of sugars was monitored by daily measurements of the °Baumé. Daily temperature controls and microbiological analyses were also performed. Fermentations took about 8 days for all wines and devatting was performed at the end. Must samples were immediately frozen and stored for molecular analyses (RFLP on lees, previously described in “Molecular analyses”).

For the malolactic fermentation, all wines were inoculated with an aliquot of commercial *Oenococcus oeni* bacteria (Viniflora Oenos, Chr Hansen) following manufacturer's instructions. At the end of the malolactic fermentation, potassium metabisulfite was added (0.06 g/l). Wine samples were collected before and after the malolactic fermentation for downstream oenochemical analyses. After racking, potassium metabisulfite was added (0.06 g/l) and the wines were bottled the following December. This was enough time to decant thin lees.

During fermentation, must samples were taken every day and diluted in sterile peptone water (0.1% Bacteriological Peptone, Oxoid). Samples were inoculated in duplicate in WL Nutrient Agar and Lysine Agar (Oxoid) [19]. Additional microbiological analyses on WL Nutrient Agar and Lysine Agar (Oxoid) and on Tomato Juice Agar (Fluka) were performed just before bottling to ensure no extraneous microorganisms could proliferate and alter the bouquet of the wines [19].

Alcohol content, density, pH, total acidity, volatile acidity, reducing sugars, total and free sulfurous anhydride, net extracts, total polyphenols (TPFs) content, total anthocyans, total flavonoids and chromatic features (intensity and hue) of the wines were measured according to official EU regulations [30]. Malic acid, lactic acid, succinic acid, citric acid and glycerol were measured using the reagents provided in the Diffchamb and Boehringer kits following manufacturer's instructions. Yeast available nitrogen (YAN) was measured according to [31].

Wine tasting trials of the 2004 “Nero d'Avola” (March 2005, June 2006) were conducted by a panel of 7 judges (chosen from IRVV technical personnel and outside experts). Wines were evaluated according to a 1 to 20 ranking score for their visual (0 to 4 points), olfactory (0 to 4 points) and gustatory complexions (0 to 12 points).

For volatile extraction (HS-SPME), a 40 ml vial was filled with 20 ml of sample. Extraction was performed by SPME using a DVB/CAR/PDMS fiber of 50/30 µm film thickness (Supelco, Bellefonte, PA, USA). Qualitative and quantitative analyses by GC/MS were performed as previously reported [32]–[34]. Odor thresholds were defined as in [35]–[42].

Sensory profiles [43] of the 2006 “Nero d'Avola” and “Frappato” wines were defined by two panels of trained judges [44] between 20 and 23 years of age, in different sessions (Nero d'Avola: November 22^nd^ and 29^th^ 2007 and December 06^th^ 2007; Frappato: December 19^th^, 20^th^ and 21^st^ 2007). The “Frappato” panel consisted of 9 judges (3 males, 6 females) and the “Nero d'Avola” panel of 13 judges (5 males, 8 females). During the preliminary sessions, the judges selected two sets of descriptors on the basis of the frequency (%) of their citation: 10 descriptors for the “Frappato” and 12 descriptors for the “Nero d'Avola”. The sensory profile of the “Nero d'Avola” wines was defined on the basis of 12 attributes: 2 referring to the visual aspect (red color intensity and purple reflexes), 7 to the aroma [fruity, citrus, berries (blackberry, blueberry, raspberry), cherry, dried fruit, floral, vegetative/herbaceous], 2 to the taste (acid and bitter) and 1 for oral perception (astringent). The sensory profile of “Frappato” wines was defined by 10 attributes: 2 for the visual aspect (color intensity and reflexes), 5 for aroma (fruity, vegetative/herbaceous, spicy, phenolic, microbiological), 2 for the taste (acid and bitter) and 1 for oral perception (astringent). All tests were performed between 10.00 and 12.00 a.m.. Judges sat in individual booths [44], [45] provided with white light illumination. 50 ml of each wine were served at 22±1°C (room temperature) in glasses [46] labeled with a 3-digit code and covered to prevent volatile loss. Wine descriptors were quantified using a 9-point intensity scale [47] with 1 as the lowest score and 9 as the highest. At each session wines were evaluated in triplicate and presented to the judges in a random order. A total of twelve sample were prepared for each session. Water was provided for rinsing between wines. All data were recorded using FIZZ software (FIZZ version 2.20H, Couternon, France).

## Results

### Natural populations of Saccharomyces yeasts in south-eastern Sicily (Italy) wineries: molecular characterization and population studies

The aim of the present study was to identify yeast strains of industrial interest and assess the economic potential of yeasts populations occurring in areas where wine fermentation is still performed using traditional stone-concrete troughs. Therefore, late fermentation Nero d' Avola must samples were collected from the wineries indicated in figure 1 (letters from A to G), during the 2002 and the 2003 harvests and 918 colonies of the *Saccharomyces* genus were isolated (see methods).

The study took several years to complete. At the time we began the characterization of our isolates (2002) we made use of the molecular techniques currently available. In particular, the analysis of restriction fragment length polymorphism patterns of mitochondrial DNA (mt-DNA RFLP) obtained using the *RsaI* enzyme [12] and the analysis of PCR amplification patterns of δ nuclear interspersed sequences (δ-PCR) obtained using the δ1–δ2 primer couple [13].

Although both techniques have since been improved, and a higher resolution power has been achieved (using the *HinfI* enzyme for mtDNA RFLP and the δ12-δ2 primer couple for δ-PCR [26], [48]), we completed our analysis using the same methodology with which we had started. Therefore we obtained a conservative estimate of the level of diversity (number of strains) present in our collection of isolates.

Based on the mt-RFLP patterns, the 352 isolates of 2002 could be ascribed to 89 different strains (indicated by Roman numerals, from I_02_ to LXXXIX_02_ in table 1 and tables S1, S2, S3, and S4) and the 566 isolates of 2003 could be ascribed to 132 different strains (I_03_ to CXXXIV_03_). Twelve strains were present in both years: thus the final number of strains identified amounted to 209. These are listed in tables S1, S2 and S3 (note: strains XXXIX_03_ and XLI_03_ are absent since they were later found to be identical to others already present in the tables).

**Table 1:** Frequencies^1^ of the strains that were most abundant (≥1%) and/or were present in both the 2002 and 2003 vintages.

2003 strains	Frequencies in 2003	2002 strains^2^	Frequencies in 2002	Frequency variation (%2003-%2002)
I_03_	15.55	XXIII_02_	14.20	+1.35
IV_03_	10.95	XIII_02_	12.50	−1.55
V_03_	9.01	IX_02_	7.67	+1–.34
XIII_03_	5.65	XI_02_	4.26	+1.39
LXXIV_03_	6.01	-	-	+6.01
XVIII_03_	4.24	VIII_02_	4.55	−0.31
VII_03_	3.18	XXII_02_	1.99	+1.19
II_03_	2.83	VII_02_	1.70	+1.13
CXXII_03_	2.12	-	-	+2.12
XI_03_	1.94	LIX_02_	4.55	−2.61
VI_03_	1.77	LXVI_02_	0.85	+0.92
LXXXVI_03_	1.41	XXVII_02_	0.57	+0.84
XV_03_	1.24	-	-	+1.24
CVI_03_	1.06	-	-	+1.06
XXVI_03_	1.06	-	-	+1.06
XCVII_03_	0.71	LXXIV_02_	0.57	+0.14
CIV_03_	0.36	XXVIII_02_	0.28	−0.70
-	-	XLI_02_	10.80	−10.80
-	-	XVII_02_	2.57	−2.56
-	-	I_02_	2.27	−2.27
-	-	XX_02_	1.70	−1.70
-	-	XV_02_	1.42	−1.42

1 Percent of isolates with the indicated mt-DNA RFLP pattern, over the total number of isolates.

2 strains with the same mt-RFLP pattern in 2002 and 2003, are in the same row.

We also analyzed strain distribution by taking into consideration: i) the strains present in each fermentation; ii) the total number of strains present in the 2002 and 2003 harvests separately; iii) the variations in the distribution of the strains between the 2002 and the 2003 harvests.

- Each fermentation showed the presence of a number of different strains, with relative ratios differing from sample to sample. We calculated the frequency of each strain, as the percentage of isolates with the same mt-DNA RFLP pattern, over the total number of isolates. In many fermentations the most frequent strain was found in less than 25% of cases; however in a few fermentations one strain was significantly more frequent than the others (e.g. XLI_02_ had a frequency of 75.5% in D1 and I_03_ had a frequency of almost 83% in B5, table S1).
- When considering the entire 2002 vintage (table S2), only three strains had a frequency higher than 10% (XIII_02_, XXIII_02_ and XLI_02_), while the frequency of most of the others was less than 1%. A similar situation was found in 2003 (table S3) with only two strains having frequencies higher than 10% (I_03_ and IV_03_) and most of the others with less than 1%.
- 12 strains were present in both years but only few of them maintained their frequency. A common strain of 2002 (XLI_02_) had completely disappeared in 2003 while new strains were found in 2003, some with a relatively high frequency (e.g. LXXIV_03_). 17 strains found in the 2002 vintage and 24 found in the 2003 vintage were common to samples coming from two or more sites, while all other strains were found only in one site (table 1 and tables S2 and S3).

The patterns of the strains identified during the 2002 and 2003 vintages were compared to those of five commercial yeast strains which were used in two cooperative wine growers' associations and in three private wineries located in the same territory, to check for contamination (which could have occurred *via* insects, vehicles or people). No similarities could be found.

Next, we analyzed the δ-PCR patterns of the isolates with identical mt-DNA RFLP patterns (781 isolates in 72 groups), identifying 247 different variant patterns for the interdelta sequence (delta patterns, table S4). The most frequent pattern (considering both vintages), was found in 11% of the isolates (100 isolates in ten samples from four different wineries: A, B, E and F). 13 patterns (a total of 228 isolates) were found in 1–3% of cases. All the other patterns were present at frequencies below 1%.

### Phenotypic characterization of the strains

**Figure 2:**
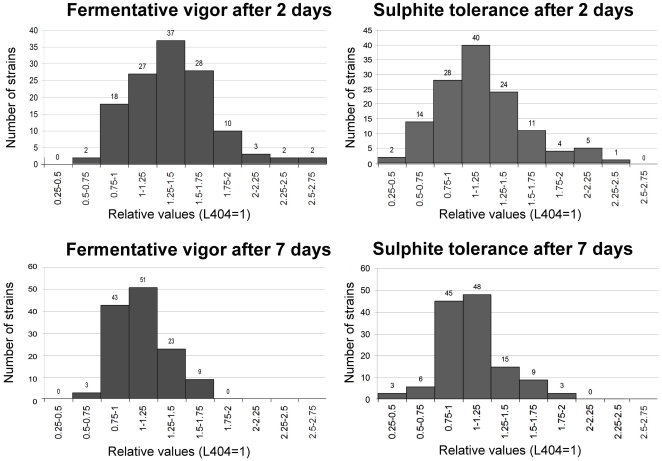
Analysis of fermentation vigor (top) and sulfite tolerance (bottom) after two days (left) and seven days (right) of 129 *Saccharomyces* strains isolated from spontaneous fermentations. A large number of isolates show values higher than the control strain L404.

**Figure 3:**
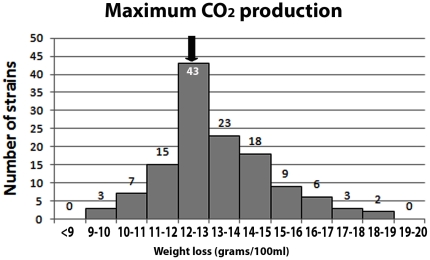
CO_2_ production (as derived from the measured weight loss) of 129 *Saccharomyces* strains, upon 300 g/l sugar supplementation. The weight loss of L404 is indicated by an arrow (12.48 g). The number of strains per each frequency class is indicated.

129 strains were assayed for fermentation vigor and sulfite tolerance at 2 and 7 days. The results of these analyses are shown in figure 2, where values are given as ratios to the control strain L404 (L404 = 1). Similar results were obtained when the performance of these strains was compared with that of the Zymaflore F10 commercial yeast strain (data not shown). For the L404 strain, average fermentation vigor values (measured as gCO_2_/100 ml) were 1.4 after 2 days and 7.7 after 7 days, and sulfite tolerance values were 1.4 after 2 days and 8.7 after 7 days. Although L404 is considered a good oenological starter, 39 strains showed levels of all four parameters higher than those of the control strain L404. Finally we monitored musts' weight loss (due to CO_2_ production) upon supplementation of glucose (an indication of fermentation power) in comparison with that of musts fermented with the L404 strain. In this latter case an average weight loss of 12.48 g was measured (fig. 3). Among our isolates, 87 had higher weight loss values.

We also monitored growth patterns [1], observing only suspension and flocculation (as is most common in nature). 12% of the strains showed flocculation, a frequency higher than previously reported (6% [49]). No strain was found to produce foam. All strains were capable of producing spores, although to different degrees. In all cases ascii were found to contain 2–4 spores as expected for the *Saccharomyces* genus [50].

Microbiological analyses were performed to determine the production of acetic acid (assessed by CaCO_3_ solubilization). Only 15 strains were found to produce high levels of acetic acid, while the remaining 114 showed average or low production levels. Tests performed on BiGGY Agar identified 15 strains producing very low levels of H_2_S, 21 producing low levels, 74 producing average levels and 19 producing high levels. Tests performed with arbutin showed no β-glucosidase activity, confirming that this feature is rare in the *Saccharomyces* genus [51].

### Preliminary fermentation studies, selection and molecular characterization of the B2–25 and B2–48 isolates

**Figure 4:**
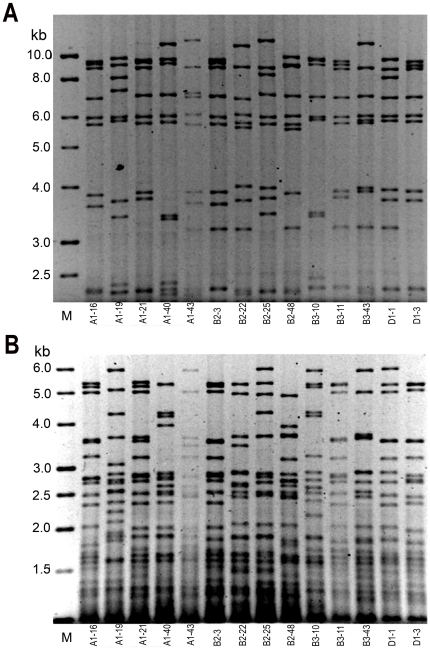
Mt-DNA RFLP patterns of 14 isolates (indicated) used for the 100 liter fermentations of 2004. A. Patterns obtained with the *RsaI* enzyme. B. patterns obtained with the *HinfI* enzyme. M, molecular marker (1 kb ladder, BHD).

Of all the strains analyzed, 28 with the best fermentation and technological features (fermentative vigor, SO_2_ tolerance, maximum CO_2_ production, low H_2_S and acetic acid production, suspended growth pattern) were selected as starters for small-scale fermentations (in 1 liter of “Nero d'Avola” sterile must) after the 2003 harvest. Samples inoculated with the *S. cerevisiae* Zymaflore F10 Laffort strain and non-inoculated samples were also prepared for comparison. The progress of each fermentation was monitored measuring weight loss due to CO_2_ production. At the end of each fermentation several parameters were assessed, including levels of alcohol, volatile acids, acetaldehyde, anthocyans and flavonoids (data not shown). Eventually 14 strains (isolates: A1–16, A1–19, A1–21, A1–40, A1–43, B2–3, B2–22, B2–25, B2–48, B3–10, B3–11, B3–43, D1-1 and D1–3) were chosen and used as starters for fermentations in 100 liters of “Nero d'Avola” must in 2004. In addition to the *RsaI* mt-DNA RFLP pattern analyses, *HinfI* mt-DNA RFLP pattern analyses were performed on these isolates to further confirm their genetic diversity (fig. 4). During fermentation, daily controls were performed to monitor the proliferation of each isolate within the microbiological flora of each must (figure S1), and at the end of each fermentation to assess the microbiological stability of the wines. Oenochemical analyses were performed at the end of the process to assess the impact of each starter isolate on each fermentation. Comparisons were again made with musts inoculated with Zymaflore F10 Laffort and with non-inoculated musts. The ability of each isolate to carry on the entire fermentation was confirmed by comparing the mt-DNA RFLP from the lees collected at the end of fermentation with those of their pure cultures (figure S2).

The 14 wines produced were tasted by a panel of oenologists 3 months after bottling (March 2005, fig. 5). All the wines scored better than the wine obtained from non-inoculated must. Six of them (fermented by the A1–21, B2–25, B2–48, B3–10, B3–11, B3–43 isolates) scored better than the Zymaflore F10 Laffort wine. Wines B3–11, B2–25, B2–48 and B3–43 received an overall score above 16/20. After bottle ageing and further tasting (June 2006, data not shown), A1–21, B2–25 and B2–48 wines were found to be the best of all the wines produced. The A1–21 isolate was selected for industrial production and it is currently used as a fermentation starter worldwide (NDA21 [52], [53]).

**Figure 5:**
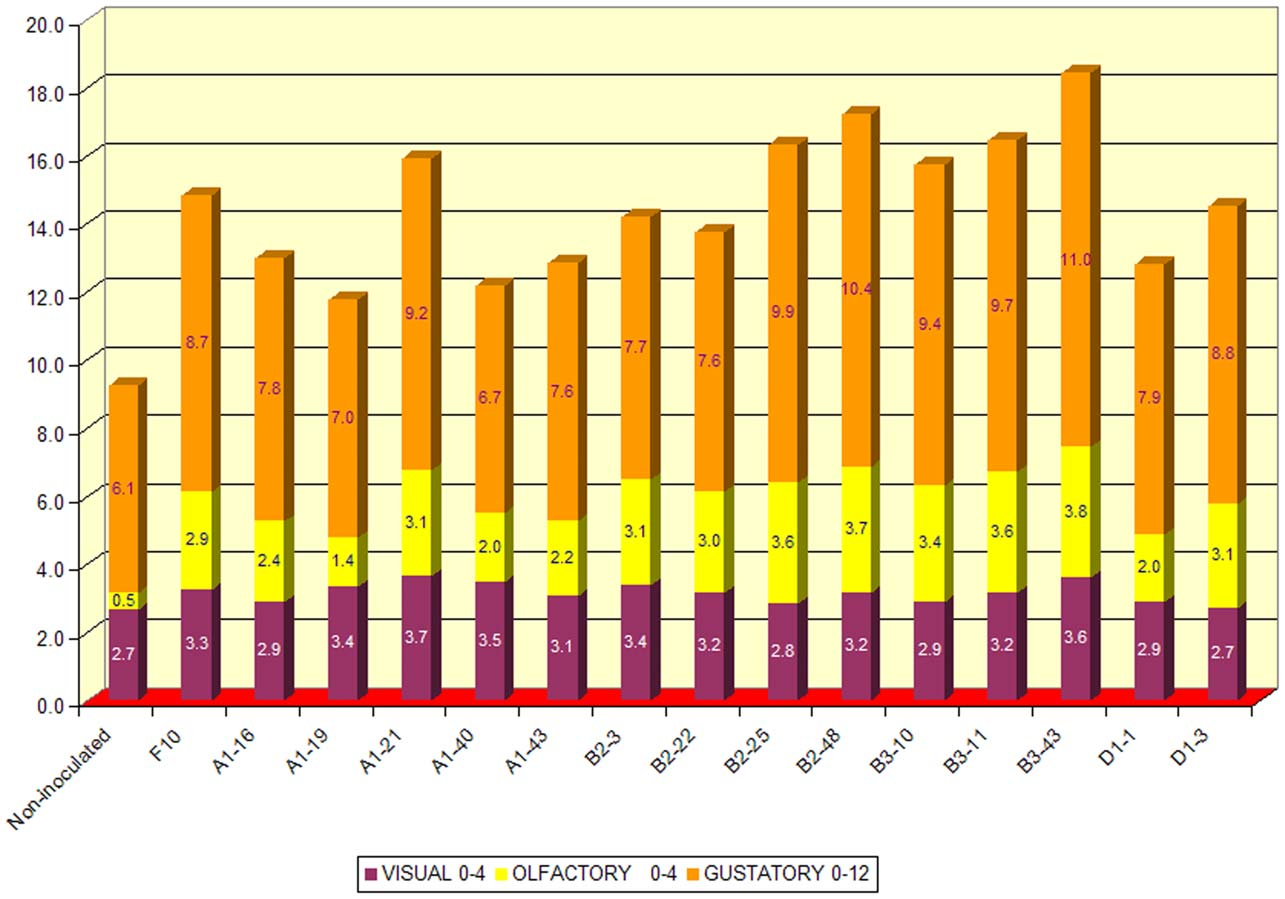
Wine tasting results for the 14 experimental wines. The name of each starter is indicated.

The results obtained prompted us to proceed in our investigation, focusing on the B2–25 and B2–48 isolates. However before starting new fermentations, we performed two additional molecular analyses to be able to definitely ascribe these isolates to the *S. cerevisiae* species. This was confirmed in both cases, by amplifying the ITS ribosomal DNA region (see methods; fig. S3).

### B2–25 and B2–48 100 liter fermentations and analysis of the wines

During the 2006 harvest, 100 liters of “Nero d'Avola” and “Frappato” musts were inoculated with pure cultures of the B2–25 and B2–48 isolates, in a winery environment. For comparison, 100 liter must aliquots were also inoculated with two commercial yeast strains widely utilized by the Sicilian wine industry, ICV-D254 and QD145.

**Figure 6:**
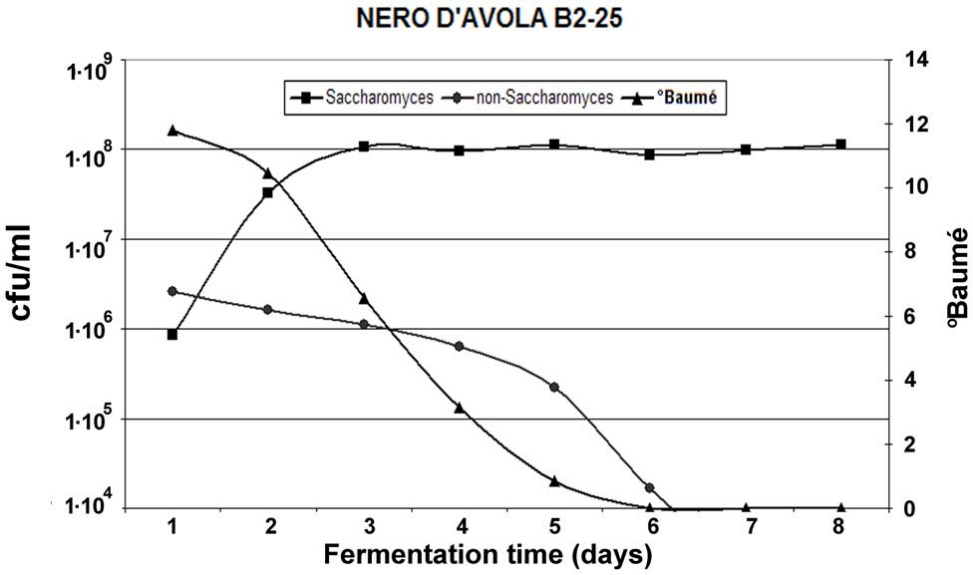
Growth curves of *Saccharomyces* and non-*Saccharomyces* yeasts in B2–25-inoculated Nero d'Avola must. The relative sugar consumption (expressed as °Baumé) is indicated.

**Figure 7:**
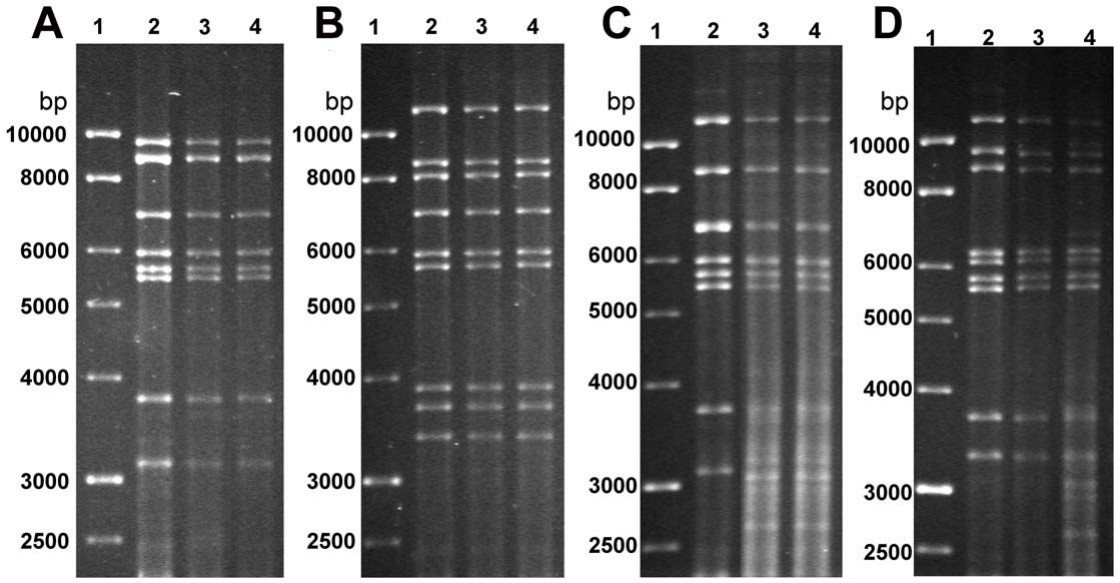
Mt-DNA RFLP patterns obtained at the end of fermentation from Nero d'Avola (lane 3) and Frappato (lane 4), inoculated with B2–48 (A), B2–25 (B), ICV-D254 (C) and QD-145 (D). Control RFLP patterns obtained with DNA from the pure cultures of each isolate are shown in lane 2 of each panel. Lane 1, molecular marker (1 kb ladder, BDH).

Daily microbiological analyses demonstrated that each isolate rapidly predominated in the microbiological flora of the musts. The results of the analyses performed on “Nero d'Avola” musts inoculated with B2–25 are shown in figure 6. Similar results were obtained on “Frappato” musts and with the B2–48 isolate and the commercial strains (fig. S4). The quick onset of the exponential growth phase helped prevent the predomination of non-*Saccharomyces* species, which rapidly declined and did not significantly contribute to the fermentation process. Fermentations lasted for 8 days and the level of residual sugars in the wines was minimal at the end. Molecular analyses were performed to ensure that the mt-DNA RFLP patterns of the yeast recovered from the wines at the end of fermentation were identical to those of the isolates inoculated in the musts (fig. 7).

Yeasts affect the quality of wines in two ways. First, by transforming sugars into alcohol and CO_2_. Secondly, by producing a series of secondary metabolites which enrich wines with characteristic aromas and increase their chemical complexity [24]. To understand how each isolate contributed to the quality of the wine produced we compared musts before and after fermentation by measuring some of the most important oenochemical parameters (table 2).

**Table 2:** Oenochemical parameters measured in Nero d'Avola and Frappato pre-fermentation musts and wines (before malolactic fermentation).

Oenochemical Parameter	NdA^1^	NdA^2^+B2–25	NdA^2^+B2–48	NdA^2^+ICV-D254	NdA^2^+QD145	Frappato^3^	Frappato^4^+B2–25	Frappato+B2–48	Frappato^4^+ICV-D254	Frappato^4^+QD145
Brix	20.97					19.81				
pH	3.43	3.62	3.64	3.69	3.67	3.45	3.58	3.54	3.58	3.54
Total acid (g/l)	6.00	6.00	6.10	5.20	6.32	7.30	6.70	6.80	6.50	6.70
Malic acid (g/l)	1.47	1.88	1.99	1.24	1.64	1.74	1.63	1.60	1.26	1.31
Lactic acid (g/l)	0.00	0.05	0.03	0.11	0.06	0.03	0.03	0.03	0.03	0.03
Succinic acid (g/l)	0.00	0.71	0.88	0.73	0.75	0.06	0.58	0.72	0.59	0.72
Citric acid (g/l)	0.26	0.32	0.32	0.31	0.32	0.36	0.42	0.42	0.40	0.41
Glycerol (g/l)	0.56	8.24	7.78	7.23	8.97	0.70	7.30	8.30	7.30	7.50
YAN^5^ (mg/l)	157					216				
Alcohol %	N/A^7^	12.38	12.45	12.47	12.30	N/A	12.13	12.39	12.56	12.40
Wine density	N/A	0.9947	0.9948	0.9945	0.9943	N/A	0.9960	0.9963	0.9960	0.9960
Net extract (g/l)	N/A	27.9	28.4	26.9	27.1	N/A	29.6	30.9	30.3	30.4
Reducing sugars (g/l)	N/A	1.50	1.50	1.70	1.56	N/A	2.40	2.70	2.70	2.40
Total SO_2_ (mg/l)	N/A	19.0	16.0	22.0	21.0	N/A	30.0	29.0	30.0	30.0
Free SO_2_ (mg/l)	N/A	12.0	11.0	11.0	12.0	N/A	20.0	19.0	16.0	14.0
Volatile acidity (g/l)	N/A	0.21	0.16	0.24	0.17	N/A	0.18	0.18	0.19	0.19
TPFs^6^ (mg/l)	N/A	2273	2257	2196	2129	N/A	2013	2088	1954	1913
Color intensity	N/A	8.70	9.34	9.20	8.84	N/A	4.44	4.70	4.22	4.46
Tonality	N/A	0.616	0.602	0.663	0.667	N/A	0.857	0.872	0.909	0.906
Anthocyans (mg/l)	N/A	435	364	355	350	N/A	86	88	89	86
Tot flavonoids (mg/l)	N/A	1747	1705	1734	1648	N/A	1483	1610	1595	1474

1 Nero d'Avola must;

2 Nero d'Avola wines (the isolate used in each fermentation is indicated);

3 Frappato must;

4 Frappato wines (the isolate used in each fermentation is indicated);

5 Yeast Available Nitrogen;

6 Total Polyphenols;

7 Does not apply.

Values were generally similar in wines produced from the same musts. All starters left similar amounts of residual sugars and the final alcohol content was similar in all cases (12–12.5%, v/v). Glycerol levels varied between 7–9 g/l. At these concentrations glycerol contributes to the viscosity and smoothness of the wine and has a positive effect on taste [54]. Acidity content was similar in all wines (6–7 g/l except for Nero d'Avola ICV-D254) and the levels of volatile acidity were always low (below 0.25 g/l, mostly due to acetic acid). An increase in the synthesis of succinic acid was observed in all the wines. TPF content was considerable in all wines, with a slightly higher level in the B2–25 and B2–48 wines. These compounds are important for the organoleptic properties of a wine; in their presence, the moderate daily consumption of red wine acts to reduce cardiovascular and cancer risk factors [55].

Malolactic fermentation was performed, dramatically reducing the levels of malic acid in all wines (table S5). As expected, an increase in the amount of lactate and a decrease in the total acidity was observed in both “Nero d'Avola” and “Frappato” wines. This contributed to a general improvement in the taste of the wines.

SPME-GC-MS analyses were performed to identify and quantify the volatile compounds produced by the starter yeasts (Table S6). A number of esters were identified, the most abundant being ethyl octanoate, ethyl hexanoate, ethyl decanoate and isoamyl acetate. Among the fermentation aromas, the main compounds were isoamyl and β-phenylethyl alcohols. Among the varietal aromas, terpenes and C13 norisoprenoids were identified which have a pleasant aroma and a very low olfactory threshold and are therefore perceived during wine tasting even in low concentrations. Due to several synergic and antagonist effects, they correlate with the citrus and floral descriptor.

A different ratio of fermentation *vs.* varietal aromas can be observed when Frappato and Nero d'Avola wines are compared, which accounts for the differences in the two cultivars. Frappato wines had a greater amount of terpenes and C_13_ norisoprenoids than Nero d'Avola wines. This is in agreement with [56] who analyzed and compared the composition of the most important varieties of grape cultivated in Sicily, including Nero d'Avola and Frappato.

With regard to the Frappato wines, fermentation with the commercial yeast strains and those we isolated led to a similar volatile composition (total amount of esters, terpenes, and alcohols). An interesting difference, however, is that the B2–25 wine had a greater amount of some of the most volatile compounds: ethyl butanoate, ethyl 2-methylbutanoate, ethyl 3-methyl butanoate, isoamyl acetate and hexyl acetate. Moreover, this wine had the least amount of ethyl decanoate and diethyl succinate. These differences could have a positive effect on the wine aroma because they would increase the fruity note while reducing the grape and wine notes. Moreover a greater amount of hexyl acetate, such as that observed in B2–25 and B2–48 wines, might be desirable as this compound is considered to be a quality factor in wine [57].

Similar amounts of fermentation and varietal volatile aromas were found in all the Nero d'Avola wines. However, a greater amount of esters was found in the B2–25 wine (mainly due to ethyl decanoate and ethyl octanoate). For this sample, a greater amount of isoamyl alcohol and β-phenylethyl alcohol was also found. In particular, the amount of β-phenylethyl alcohol, which is responsible for floral notes, was two-three times higher than that of wines from the B2–48 and commercial yeasts. As far as the aroma constituents are concerned, therefore, our isolates preserved the particularities of the grape cultivars.

**Figure 8:**
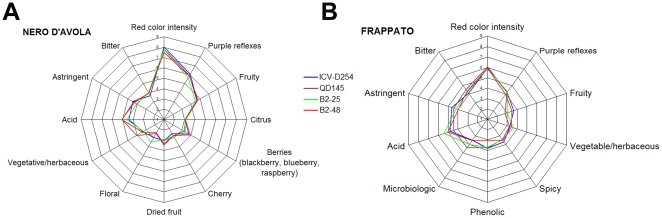
Results of the sensory analyses performed on the Nero d'Avola (A) and Frappato (B) wines. See text for further explanation.

The sensory profiles of Nero d'Avola and Frappato wines are shown in fig. 8. Among the Nero d'Avola wines, the B2–25 was the least astringent, the highest value for the vegetative/herbaceous descriptor was found in QD145, the highest intensity of dried fruit aroma was found in B2–48 and QD145, and the B2–48 and ICV-D254 wines had the strongest berries aroma (fig. 8A). Among the Frappato wines, the fruity aroma was strongest in the B2–48 and ICV-D254 wines, the B2–48 and the QD145 wines were those where the phenolic descriptor was weakest and the microbiological descriptor was the strongest, while all the wines had the same level of spicy aroma (fig. 8B). In general, the sensory profiles of the wines were similar in wines made with the same must. Thus, our isolates resulted in wines whose aromatic and sensory profiles were consistent with the cultivar of the musts, and produced wines which were comparable to those made with two well-known commercial yeast strains.

## Discussion

Overall, our study shows that an economic potential exists for the patrimony of biodiversity among the fermentation yeast populations present in south-eastern Sicily. It is important to stress that the isolates analyzed were recovered from musts fermented in traditional stone-concrete troughs following long-established fermentation practices. These practices are soon to disappear as a result of the industrialization of the fermentation process (using selected yeast strains) and because of the strict application of current EU food safety regulations [58]. Therefore our study addresses the question of the level and the value of yeast biodiversity preserved by traditional winery practices. This is a very different question than that addressed in the vast majority of studies, where grapes collected from vineyards are fermented under sterile laboratory conditions (e.g. [7], [9]). In fact, a number of studies have characterized the yeast biota present on grapes, showing that *S. cerevisiae* is rather rare and that enrichment procedures are needed to obtain isolates of this species [59]. Several authors have suggested that the *S. cerevisiae* strains have been selected within the particular niche of the winery, due to their ability to withstand the high alcohol levels found there [60]–[62].

More recently it has been shown that there are differences between the yeast communities of the wineries and that of the vineyard, even when the two environments are in physical proximity. The yeast flora of a winery is a mixture of species brought in on the grapes and others which are resident in the winery (a “perennial” component maintained over the years [4], [63], [64]). The winery environment represents an ecological niche habitat where certain yeast species are favored and persist. Different yeast components can predominate during spontaneous fermentation, depending on the equilibrium between the yeasts of perennial and vineyard origin at the start of fermentation [3], [4], [65]. However, exchanges exist between the vineyard and the winery so that the same yeast strains can be found in the two environments: commercial yeast strains in use in the winery can be transported to the vineyard and recovered from the musts of grapes fermented in the laboratory. Nevertheless, these exchanges happen within a limited range [65], [66]. Furthermore, in the area we analyzed, there might well be a limit to the extent of the exchange possible between vineyards and wineries given that the commercial strains used in some wineries present in the same area were never found in the musts fermented in the wineries we considered.

The rationale of this study, therefore, was to identify *Saccharomyces* (and *S. cerevisiae* in particular) strains that would be dominant in winery musts at a late stage of fermentation. These would be expected to possess high fermentative vigor, sulfite tolerance and fermentative power. This expectation was confirmed. Furthermore we showed that all the 14 *Saccharomyces* strains we selected in 2004 (including the two *S. cerevisiae* B2–25 and B2–48) were able to dominate the fermentation process during a fermentation conducted in a winery environment (figures 6 and 7; figures S1, S2 and S4). At the end of each fermentation, the *Saccharomyces* population was found to be homogenous with the starter inoculated. This feature is important from the industrial point of view and it is not to be taken for granted, given that a recent study in which *Saccharomyces* yeast strains were recovered from grapes collected from the same region and fermented in the cellar reported that only 50% (at best) of the *S. cerevisiae* recovered belonged to the strain of the starter inoculated [7].

Most of our yeast isolates were shown to possess technological and quality characteristics comparable or even superior to those of some yeast strains widely used by the industry. Fermentation with the B2–25 and B2–48 isolates led to the production of wines whose chemical complexity and sensory profiles were comparable to those of wines obtained with two commercial yeast strains used in the Sicilian wine industry. The characteristics of these wines were also consistent with the cultivar of the musts, indicating that the isolates chosen do not alter or impact negatively on the quality of the wines produced. Furthermore, in tasting sessions, B2–25 and B2–48 wines performed better than that made with a commercial yeast strain, highlighting the industrial potential of these two isolates.

We believe, therefore, that maintaining the biodiversity of local yeast populations, through the preservation of traditional winery practices, can be beneficial to the local wine industry and help promote local economic activities which can have an impact on the global market.

## Supporting Information

Figure S1 **Growth curves of** ***Saccharomyces*** **and non-** ***Saccharomyces*** **yeasts in the 2004 (100 liters) fermentations.** Starter yeast strains are indicated in each panel. The relative sugar consumption (expressed as °Baumé) is indicated. In each fermentation the growth of *Saccharomyces* yeasts reached plateau and that of non-*Saccharomyces* yeasts was reduced to negligible levels, well before the end of the process (except in the spontaneous fermentation).
(TIF)

Figure S2 **Molecular controls on the 2004 (100 liters) fermentations.** In each group of three lanes, a molecular marker (same of figure 4) is shown together with the RFLP of the mt-DNA of the lees and that of the starter pure culture (both were digested with the *RsaI* restriction enzyme).
(TIF)

Figure S3 **B2–25 and B2–48 are members of the** ***S. cerevisiae*** **species.** A. Restriction analyses of the ITS amplicons for the B2–25 (lane 1) and B2–48 (lane 2) isolates, obtained with the ITS1 and ITS4 primer pair and after digestion with the HaeIII endonuclease. B. ITS amplicons obtained with the SC1 and SC2 primer pair, on B2–25 (lane1) and B2–48 (lane2) DNA. Controls (lanes 3, *S.cerevisiae* Diproval strain 6167; lanes 4, *S.bayanus* Diproval strain 11719) are shown for comparison. M, molecular marker. Lane 5 in B, no DNA-containing sample.
(TIF)

Figure S4 **Growth curves of** ***Saccharomyces*** **and non-** ***Saccharomyces*** **yeasts in 2006 Nero d'Avola and Frappato musts.** Starter yeast strains are indicated in each panel. The relative sugar consumption (expressed as °Baumé) is indicated.
(TIF)

Table S1 Each of the 18 sheets accounts for each of the 2002 (A1, A2, A3, A4, B2, B3, D1) and 2003 (A5, A6, A7, B4, B5, B6, D2, D3, E1, F1, G1) samples. For each strain (mt-DNA polymorphism class), the table shows the corresponding isolates, the total number of isolates and the percentages of each strain in each sample. For each of the 2003 strains, the corresponding 2002 strain is also indicated.
(DOC)

Table S2 **Analysis of the 2002 vintage.** For each of the different strains (I to LXXXIX), the table shows the number of isolates for each of the samplings (A1, A2, A3, A4, B2, B3, D1), the total number of isolates per strain and the percentages of that strain in the population.
(DOC)

Table S3 **Analysis of the 2003 vintage.** For each of the different strains (I to CXXXIV), the table shows the corresponding 2002 strain, the number of isolates for each of the samplings (A5, A6, A7, B4, B5, B6, D2, D3, E1, F1, G), the total number of isolates per strain and the percentages of that strain in the population.
(DOC)

Table S4 **Delta sequence amplification pattern analyses.** All the isolates belonging to the same strain (indicated in the first two columns) are grouped according to their different delta sequence amplification patterns. Note that the groups indicated from 1 to 19 are not the same from one row to the next since the strains are different (e.g. group 1 for class I is different from group 1 for class II).
(DOC)

Table S5 **Acid content (g/l) at the end of malolactic fermentation.**
(DOC)

Table S6 **Average composition of single volatile compounds in Nero d'Avola (NdA) and Frappato wines.**
(DOCX)
