# Correction: Biodiversity of Indigenous *Saccharomyces* Populations from Old Wineries of South-Eastern Sicily (Italy): Preservation and Economic Potential

**DOI:** 10.1371/annotation/cd6e2d3b-4888-4231-a13d-6ef6170b79d6

**Published:** 2012-05-15

**Authors:** Sabina Di Maio, Giuseppe Polizzotto, Enrico Di Gangi, Giusy Foresta, Giuseppe Genna, Antonella Verzera, Antonio Scacco, Gabriele Amore, Daniele Oliva

There was an error in the affiliations of the seventh and eighth authors. The correct affiliations of Antonio Scacco are:

1 Istituto Regionale della Vite e del Vino, Palermo, Italy,

3 Dipartimento di Scienze Agrarie e Alimentari, Università di Catania, Catania, Italy,

and the correct affiliations of Gabriele Amore are:

1 Istituto Regionale della Vite e del Vino, Palermo, Italy,

4 Animal Physiology and Evolution Laboratory, Stazione Zoologica “Anton Dohrn” Napoli, Napoli, Italy.

