# Supplementary figures and images for: Biodiversity of Indigenous Saccharomyces Populations from Old Wineries of South-Eastern Sicily (Italy): Preservation and Economic Potential
Source: PLoS One. 2012 Feb 29;7(2):e30428. doi: 10.1371/journal.pone.0030428 (PMC3290603; doi:10.1371/journal.pone.0030428)

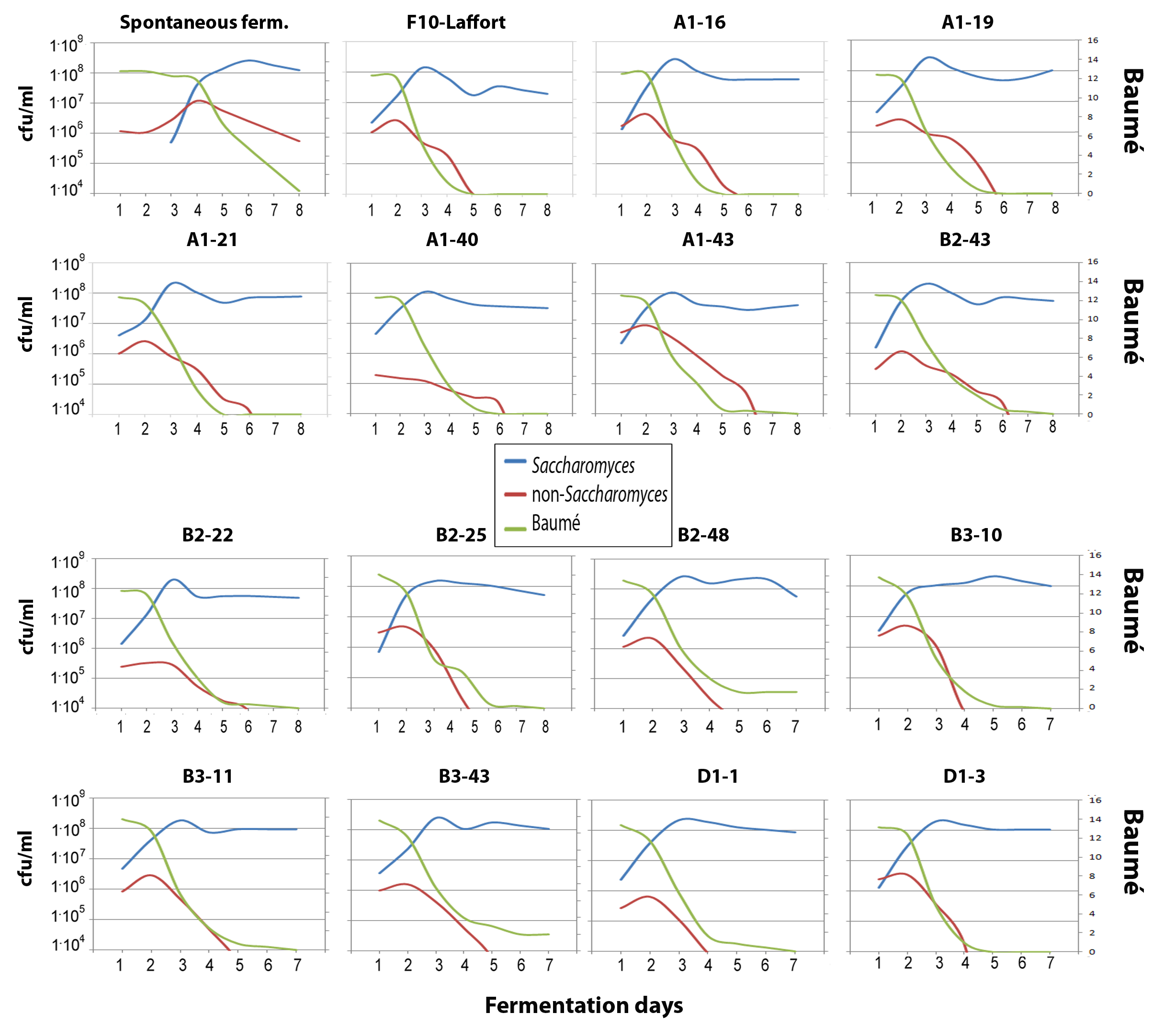

Supplement: Figure S1 — Growth curves of Saccharomyces and non- Saccharomyces yeasts in the 2004 (100 liters) fermentations. Starter yeast strains are indicated in each panel. The relative sugar consumption (expressed as °Baumé) is indicated. In each fermentation the growth of Saccharomyces yeasts reached plateau and that of non-Saccharomyces yeasts was reduced to negligible levels, well before the end of the process (except in the spontaneous fermentation). (TIF) [file pone.0030428.s001.tif]

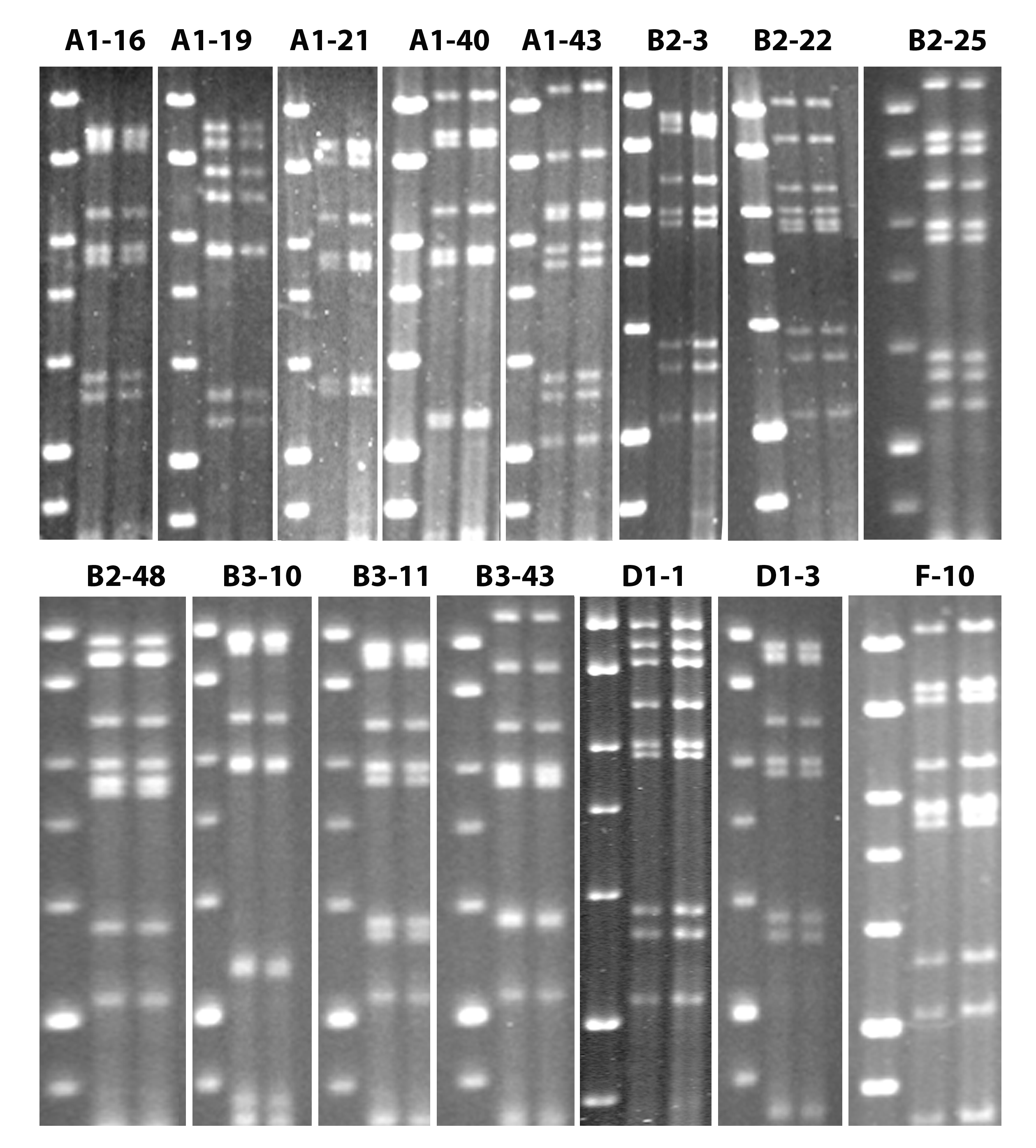

Supplement: Figure S2 — Molecular controls on the 2004 (100 liters) fermentations. In each group of three lanes, a molecular marker (same of figure 4) is shown together with the RFLP of the mt-DNA of the lees and that of the starter pure culture (both were digested with the RsaI restriction enzyme). (TIF) [file pone.0030428.s002.tif]

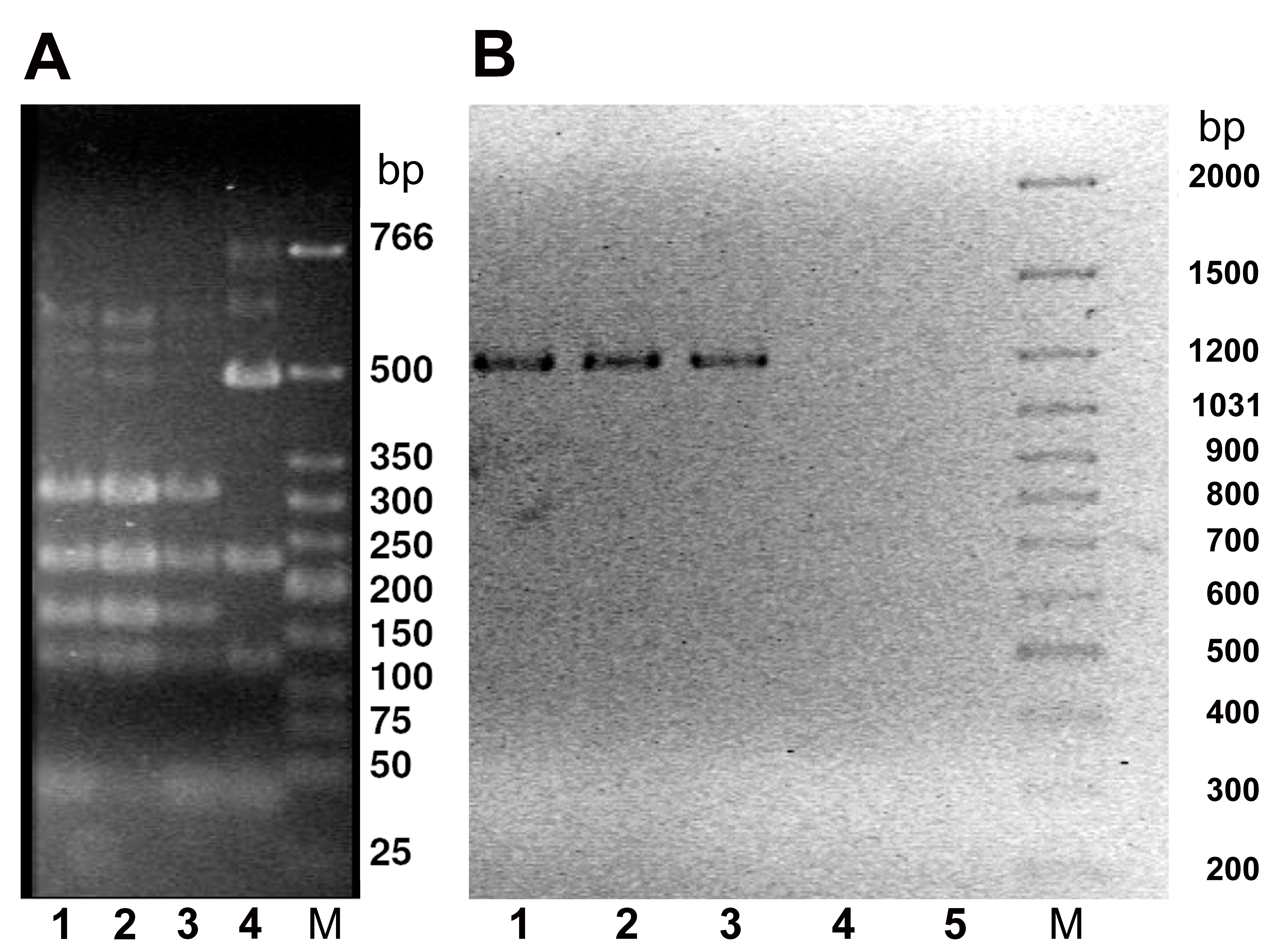

Supplement: Figure S3 — B2–25 and B2–48 are members of the S. cerevisiae species. A. Restriction analyses of the ITS amplicons for the B2–25 (lane 1) and B2–48 (lane 2) isolates, obtained with the ITS1 and ITS4 primer pair and after digestion with the HaeIII endonuclease. B. ITS amplicons obtained with the SC1 and SC2 primer pair, on B2–25 (lane1) and B2–48 (lane2) DNA. Controls (lanes 3, S.cerevisiae Diproval strain 6167; lanes 4, S.bayanus Diproval strain 11719) are shown for comparison. M, molecular marker. Lane 5 in B, no DNA-containing sample. (TIF) [file pone.0030428.s003.tif]

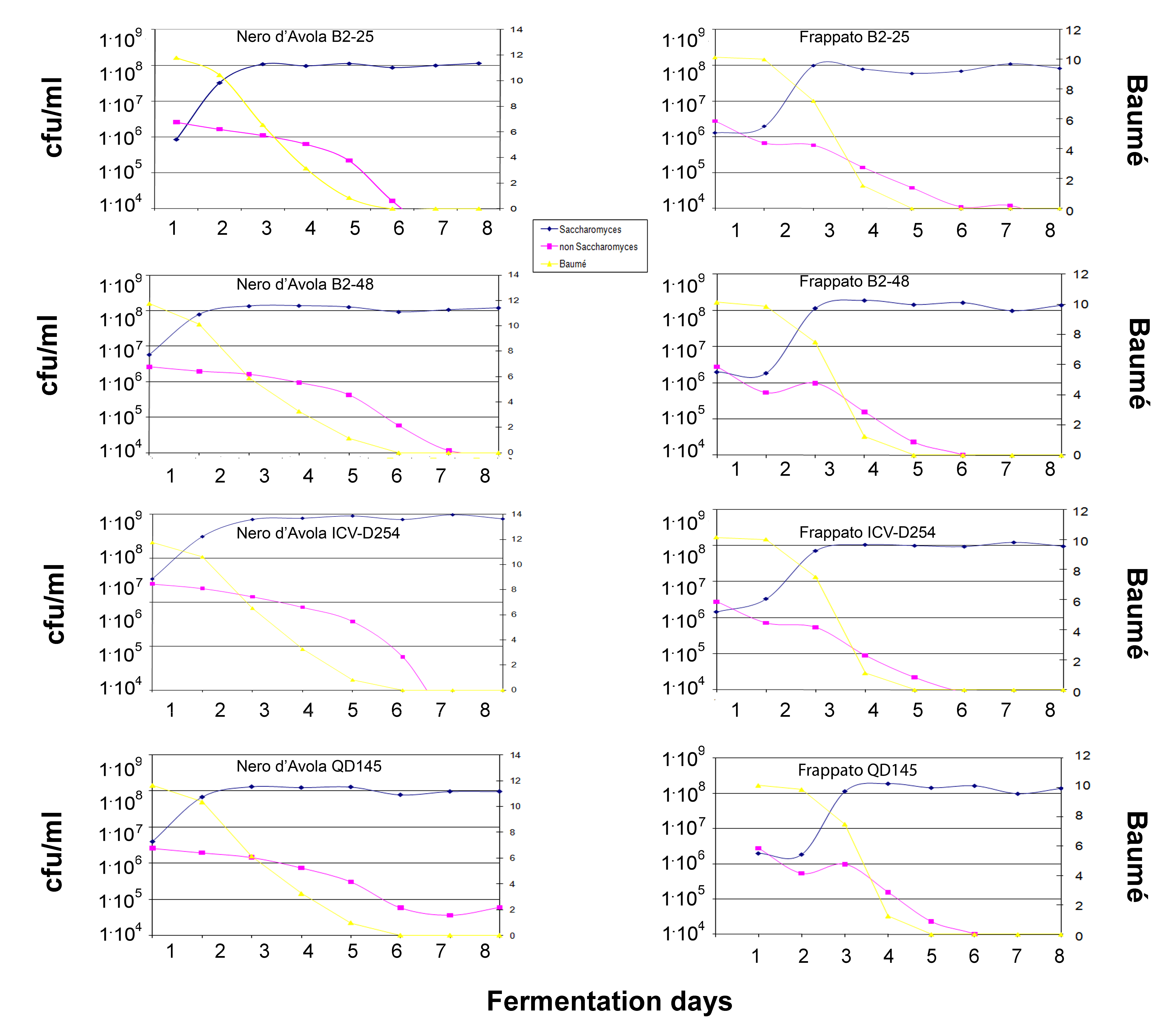

Supplement: Figure S4 — Growth curves of Saccharomyces and non- Saccharomyces yeasts in 2006 Nero d'Avola and Frappato musts. Starter yeast strains are indicated in each panel. The relative sugar consumption (expressed as °Baumé) is indicated. (TIF) [file pone.0030428.s004.tif]
