# Supplementary material for: Biodiversity of Indigenous Saccharomyces Populations from Old Wineries of South-Eastern Sicily (Italy): Preservation and Economic Potential
Source: PLoS One. 2012 Feb 29;7(2):e30428. doi: 10.1371/journal.pone.0030428 (PMC3290603; doi:10.1371/journal.pone.0030428)
Supplement: Table S1 — Each of the 18 sheets accounts for each of the 2002 (A1, A2, A3, A4, B2, B3, D1) and 2003 (A5, A6, A7, B4, B5, B6, D2, D3, E1, F1, G1) samples. For each strain (mt-DNA polymorphism class), the table shows the corresponding isolates, the total number of isolates and the percentages of each strain in each sample. For each of the 2003 strains, the corresponding 2002 strain is also indicated. (DOC) [file pone.0030428.s005.doc]

| Strains 2002 | A1 Isolates | Number of isolates | % in sample A1 |
| --- | --- | --- | --- |
| I | A1-1. A1-17. A1-28. A1-31. A1-50 | 5 | 9.6 |
| II | A1-2. A1-6 | 2 | 3.8 |
| III | A1-3 | 1 | 1.9 |
| IV | A1-4 | 1 | 1.9 |
| V | A1-5. A1-33 | 2 | 3.8 |
| VI | A1-7 | 1 | 1.9 |
| VII | A1-8. A1-44 | 2 | 3.8 |
| VIII | A1-9. A1-36. A1-47 | 3 | 5.8 |
| IX | A1-10. A1-22. A1-24. A1-34 | 4 | 7.7 |
| X | A1-11 | 1 | 1.9 |
| XI | A1-12. A1-37. A1-48 | 3 | 5.8 |
| XII | A1-13 | 1 | 1.9 |
| XIII | A1-14. A1-25 | 2 | 3.8 |
| XIV | A1-15 | 1 | 1.9 |
| XV | A1-16 | 1 | 1.9 |
| XVI | A1-18 | 1 | 1.9 |
| XVII | A1-19 | 1 | 1.9 |
| XVIII | A1-20 | 1 | 1.9 |
| XIX | A1-21. A1-39. A1-51 | 3 | 5.8 |
| XX | A1-23 | 1 | 1.9 |
| XXI | A1-26 | 1 | 1.9 |
| XXII | A1-27. A1-29. A1-30. A1-41 | 4 | 7.7 |
| XXIII | A1-32. A1-49 | 2 | 3.8 |
| XXIV | A1-35 | 1 | 1.9 |
| XXV | A1-38 | 1 | 1.9 |
| XXVI | A1-40 | 1 | 1.9 |
| XXVII | A1-42. A1-52 | 2 | 3.8 |
| XXVIII | A1-43 | 1 | 1.9 |
| XXIX | A1-45 | 1 | 1.9 |
| XXX | A1-46 | 1 | 1.9 |

| RFLP 2002 | A2 Isolates | Number of isolates | % in sample A2 |
| --- | --- | --- | --- |
| XXI | A2-1. A2-36 | 2 | 4.0 |
| XXXI | A2-2 | 1 | 2.0 |
| XV | A2-3. A2-37 | 2 | 4.0 |
| XXXII | A2-4.A2-7 | 2 | 4.0 |
| IX | A2-5. A2-9. A2-22. A2-34. A2-35. A2-39. A2-43. A2-46 | 8 | 16.0 |
| XIII | A2-6. A2-10. A2-14. A2-17. A2-21. A2-29 | 6 | 12.0 |
| VII | A2-11. A2-23. A2-26 | 3 | 6.0 |
| I | A2-12 | 1 | 2.0 |
| XXXIII | A2-13 | 1 | 2.0 |
| XXXIV | A2-16. A2-49 | 2 | 4.0 |
| VIII | A2-18. A2-19. A2-24. A2-30. A2-32. A2-44. A2-51. A2-53 | 8 | 16.0 |
| XXXV | A2-20 | 1 | 2.0 |
| XXXVI | A2-27 | 1 | 2.0 |
| XXXVII | A2-28 | 1 | 2.0 |
| XXXVIII | A2-31 | 1 | 2.0 |
| XXXIX | A2-33 | 1 | 2.0 |
| XL | A2-38 | 1 | 2.0 |
| XLI | A2-40 | 1 | 2.0 |
| XLII | A2-41 | 1 | 2.0 |
| XLIII | A2-42 | 1 | 2.0 |
| XLIV | A2-45 | 1 | 2.0 |
| XI | A2-48. A2-52 | 2 | 4.0 |
| XLV | A2-50 | 1 | 2.0 |
| XLVI | A2-54 | 1 | 2.0 |

| RFLP 2002 | A3 Isolates | Number of isolates | % in sample A3 |
| --- | --- | --- | --- |
| XIII | A3-1. A3-5. A3.7. A3-9. A3-18. A3-19. A3-20. A3-27. A3 36. A3-40. A3-42. A3-50 | 12 | 23.5 |
| XLVII | A3-2 | 1 | 2.0 |
| XXIII | A3-3. A3-10. A3-16. A3-21. A3-24. A3-28. A3-29. A3-31. A3-38. A3-41. A3-43. A3-44. A3-46 | 13 | 25.5 |
| XVII | A3-4 | 1 | 2.0 |
| XXII | A3-6 | 1 | 2.0 |
| XLVIII | A3-8 | 1 | 2.0 |
| IX | A3-11. A3-37 | 2 | 3.9 |
| IL | A3-12. A3-15 | 2 | 3.9 |
| L | A3-13. A3-30 | 2 | 3.9 |
| VIII | A3-14. A3-25. A3-48 | 3 | 5.9 |
| LI | A3-17 | 1 | 2.0 |
| XI | A3-22. A3-35. A3-49 | 3 | 5.9 |
| XX | A3-23 | 1 | 2.0 |
| LII | A3-26 | 1 | 2.0 |
| LIII | A3-32 | 1 | 2.0 |
| LIV | A3-33 | 1 | 2.0 |
| LV | A3-34 | 1 | 2.0 |
| LVI | A3-39. A3-45 | 2 | 3.9 |
| LVII | A3-47 | 1 | 2.0 |
| VII | A3-51 | 1 | 2.0 |

| RFLP 2002 | A4 Isolates | Number of isolates | % in sample A4 |
| --- | --- | --- | --- |
| L | A4-1. A4-3 | 2 | 4.0 |
| XVII | A4-2. A4-6. A4-17. A4-24. A4-27. A4-32. A4-52 | 7 | 14.0 |
| XXIII | A4-4. A4-10. A4-11. A4-12. A4-13. A4-18. A4-19. A4-20. A4-21. A4-22. A4-23. A4-28. A4-30. A4-33. A4-37. A4-39. A4-40. A4-43. A4-44. A4-46. A4-47. A4-51 | 22 | 44.0 |
| XIII | A4-5. A4-16. A4-25. A4-36. A4-38 | 5 | 10.0 |
| LVIII | A4-7 | 1 | 2.0 |
| IX | A4-8 | 1 | 2.0 |
| XLIII | A4-9 | 1 | 2.0 |
| XXII | A4-14 | 1 | 2.0 |
| LIX | A4-15. A4-35. A4-48 | 3 | 6.0 |
| XI | A4-26. A4-29 | 2 | 4.0 |
| XX | A4-34 | 1 | 2.0 |
| LX | A4-41 | 1 | 2.0 |
| LXI | A4-42 | 1 | 2.0 |
| LXII | A4-45 | 1 | 2.0 |
| LXIII | A4-50 | 1 | 2.0 |

| RFLP 2002 | B2 Isolates | Number of isolates | % in sample B2 |
| --- | --- | --- | --- |
| LXIV | B2-1 | 1 | 2.0 |
| I | B2-2 | 1 | 2.0 |
| LIX | B2-3. B2-8. B2-18. B2-21 | 4 | 8.0 |
| LXV | B2-4 | 1 | 2.0 |
| XIII | B2-5. B2-6. B2-9. B2-12. B2-17. B2-20. B2-26. B2-30. B2-34. B2-35. B2-38. B2-49. B2-50 | 13 | 26.0 |
| XXIII | B2-7. B2-16. B2-45. B2-47 | 4 | 8.0 |
| LXVI | B2-10. B2-13. B2-27 | 3 | 6.0 |
| LXVII | B2-11 | 1 | 2.0 |
| XI | B2-14. B2-19. B2-39 | 3 | 6.0 |
| IX | B2-15. B2-23. B2-29. B2-36. B2-41. B2-43 | 6 | 12.0 |
| XX | B2-22. B2-28 | 2 | 4.0 |
| XXV | B2-24 | 1 | 2.0 |
| LXVIII | B2-25. B2-31. B2-32 | 3 | 6.0 |
| LXIX | B2-33 | 1 | 2.0 |
| LXX | B2-37 | 1 | 2.0 |
| XV | B2-40 | 1 | 2.0 |
| LXXI | B2-42 | 1 | 2.0 |
| LXXII | B2-44 | 1 | 2.0 |
| XLIII | B2-46 | 1 | 2.0 |
| XLIV | B2-48 | 1 | 2.0 |

| RFLP 2002 | B3 Isolates | Number of isolates | % in sample B3 |
| --- | --- | --- | --- |
| XXIII | B3-1. B3-2. B3-12. B3-15. B3-20. B3-26. B3-27. B3-29. B3-31 | 9 | 18.0 |
| LXXIII | B3-3. B3-25 | 2 | 4.0 |
| LXXIV | B3-4. B3-28 | 2 | 4.0 |
| VIII | B3-5. B3-32 | 2 | 4.0 |
| LXXV | B3-6 | 1 | 2.0 |
| XX | B3-7 | 1 | 2.0 |
| XIII | B3-8. B3-16. B3-21. B3-40. B3-45 | 5 | 10.0 |
| LIX | B3-9. B3-11. B3-19. B3-23. B3-35. B3-39. B3-46. B3-48 | 8 | 16.0 |
| LXXVI | B3-10 | 1 | 2.0 |
| IX | B3-13. B3-18. B3-24. B3-33. B3-34. B3-38 | 6 | 12.0 |
| XLVII | B3-14 | 1 | 2.0 |
| LXXVII | B3-17 | 1 | 2.0 |
| LXIX | B3-22 | 1 | 2.0 |
| LXXVIII | B3-30 | 1 | 2.0 |
| LXXIX | B3-36 | 1 | 2.0 |
| LVII | B3-37 | 1 | 2.0 |
| LXXX | B3-41 | 1 | 2.0 |
| LXXXI | B3-42 | 1 | 2.0 |
| XI | B3-43. B3-44 | 2 | 4.0 |
| LXXXII | B3-47 | 1 | 2.0 |
| XXII | B3-49 | 1 | 2.0 |
| LXXXIII | B3-50 | 1 | 2.0 |

| RFLP 2002 | D1 Isolates | Number of isolates | % in sample D1 |
| --- | --- | --- | --- |
| XIII | D1-1 | 1 | 2.0 |
| XV | D1-2 | 1 | 2.0 |
| LXXXIV | D1-3 | 1 | 2.0 |
| LXXXV | D1-4. D1-36 | 2 | 4.1 |
| XLI | D1-5. D1-6. D1-7. D1-8. D1-9. D1-10. D1-11. D1-12. D1-13. D1-16. D1-17. D1-18. D1-20. D1-22. D1-23. D1-24. D1-25. D1-26. D1-27. D1-28. D1-29. D1-30. D1-31. D1-32. D1-33. D1-34. D1-37. D1-38. D1-39. D1-41. D1-44. D1-45. D1-46. D1-47. D1-48. D1-49. D1-50 | 37 | 75.5 |
| LIX | D1-14 | 1 | 2.0 |
| LXXXVI | D1-15 | 1 | 2.0 |
| LXXXVII | D1-19 | 1 | 2.0 |
| LXXXVIII | D1-21 | 1 | 2.0 |
| XXI | D1-35 | 1 | 2.0 |
| LXXXIX | D1-42 | 1 | 2.0 |
| I | D1-43 | 1 | 2.0 |

| RFLP 2003 | A5 Isolates | Number of isolates | % in sample A5 | RFLP 2002 |
| --- | --- | --- | --- | --- |
| I | A5-1. A5-8. A5-9. A5-11. A5-17. A5-41 | 6 | 11.76 | XXIII |
| II | A5-2. A5-7 | 2 | 3.92 | VII |
| III | A5-3. A5-6 | 2 | 3.92 |  |
| IV | A5-4. A5-19. A5-24. A5-31. A5-33. A5-39. A5-42. A5-44. A5-52 | 9 | 17.65 | XIII |
| V | A5-5. A5-14. A5-23. A5-28. A5-36 | 5 | 9.80 | IX |
| VI | A5-10. A5-47. A5-50 | 3 | 5.88 | LXVI |
| VII | A5-12 | 1 | 1.96 | XXII |
| VIII | A5-13 | 1 | 1.96 |  |
| IX | A5-15 | 1 | 1.96 |  |
| X | A5-16 | 1 | 1.96 |  |
| XI | A5-18 | 1 | 1.96 | LIX |
| XII | A5-20 | 1 | 1.96 |  |
| XIII | A5-21. A5-30. A5-45 | 3 | 5.88 | XI |
| XIV | A5-22 | 1 | 1.96 |  |
| XV | A5-25 | 1 | 1.96 |  |
| XVI | A5-26 | 1 | 1.96 |  |
| XVII | A5-27 | 1 | 1.96 |  |
| XVIII | A5-29 | 1 | 1.96 | VIII |
| XIX | A5-34. A5-37 | 2 | 3.92 |  |
| XX | A5-35 | 1 | 1.96 |  |
| XXI | A5-38 | 1 | 1.96 |  |
| XXII | A5-40 | 1 | 1.96 |  |
| XXIII | A5-43 | 1 | 1.96 |  |
| XXIV | A5-46 | 1 | 1.96 |  |
| XXV | A5-48 | 1 | 1.96 |  |
| XXVI | A5-49 | 1 | 1.96 |  |
| XXVII | A5-51 | 1 | 1.96 |  |

| RFLP 2003 | A6 Isolates | Number of isolates | % in sample A6 | RFLP 2002 |
| --- | --- | --- | --- | --- |
| IV | A6-1. A6-3. A6-4. A6-7. A6-9. A6-12. A6-15. A6-16. A6-34. A6-35. A6-38. A6-40. A6-42. A6-44. A6-50 | 15 | 28.85 | XIII |
| XXVIII | A6-2 | 1 | 1.92 |  |
| XXIX | A6-5 | 1 | 1.92 |  |
| XIII | A6-6. A6-26. A6-30. A6-43. A6-48. A6-52 | 6 | 11.54 | XI |
| XIX | A6-8 | 1 | 1.92 |  |
| XXX | A6-10 | 1 | 1.92 |  |
| XX | A6-11. A6-20 | 2 | 3.85 |  |
| XXXI | A6-13. A6-22 | 2 | 3.85 |  |
| VII | A6-14. A6-24. A6-25. A6-37. A6-45 | 5 | 9.62 | XXII |
| XXXII | A6-17. A6-18 | 2 | 3.85 |  |
| XXXIII | A6-19 | 1 | 1.92 |  |
| XVIII | A6-21. A6-29. A6-39. A6-49 | 4 | 7.69 | VIII |
| XXXIV | A6-23 | 1 | 1.92 |  |
| XXXV | A6-27 | 1 | 1.92 |  |
| I | A6-28. A6-32 | 2 | 3.85 | XXIII |
| XXXVI | A6-31 | 1 | 1.92 |  |
| XXXVII | A6-33 | 1 | 1.92 |  |
| XXXVIII | A6-36 | 1 | 1.92 |  |
| XV | A6-41 | 1 | 1.92 |  |
| V | A6-46 | 1 | 1.92 | IX |
| XL | A6-47 | 1 | 1.92 |  |
| XI | A6-51 | 1 | 1.92 | LIX |

| RFLP 2003 | A7 Isolates | Number of isolates | % in sample A7 | RFLP 2002 |
| --- | --- | --- | --- | --- |
| I | A7-1. A7-25. A7-26. A7-31. A7-45. A7-48 | 6 | 11.76 | XXIII |
| XLII | A7-2 | 1 | 1.96 |  |
| XLIII | A7-3. A7-7. A7-20. A7-41. | 4 | 7.84 |  |
| XLIV | A7-4 | 1 | 1.96 |  |
| XLV | A7-5 | 1 | 1.96 |  |
| II | A7-6. A7-47 | 2 | 3.92 | VII |
| XV | A7-8. A7-37 | 2 | 3.92 |  |
| XLVI | A7-9 | 1 | 1.96 |  |
| XVIII | A7-10. A7-13. A7-22. A7-28. A7-52 | 5 | 9.80 | VIII |
| IV | A7-11. A7-12. A7-14. A7-18. A7-21. A7-24. A7-30. A7-39. A7-40. A7-42. A7-43. A7-44 | 12 | 23.53 | XIII |
| VII | A7-15. A7-16. A7-29. A7-34 | 4 | 7.84 | XXII |
| XI | A7-17 | 1 | 1.96 | LIX |
| XLVII | A7-19. A7-36 | 2 | 3.92 |  |
| XXXI | A7-23 | 1 | 1.96 |  |
| XIII | A7-32. A7-50 | 2 | 3.92 | XI |
| V | A7-33 | 1 | 1.96 | IX |
| XLVIII | A7-35 | 1 | 1.96 |  |
| IL | A7-38 | 1 | 1.96 |  |
| XIX | A7-46 | 1 | 1.96 |  |
| L | A7-49 | 1 | 1.96 |  |
| LI | A7-51 | 1 | 1.96 |  |

| RFLP 2003 | B4 Isolates | Number of isolates | % in sample B4 | RFLP 2002 |
| --- | --- | --- | --- | --- |
| LII | B4-1 | 1 | 2.00 |  |
| V | B4-2. B4-8. B4-10. B4-13. B4-14. B4-16. B4-19. B4-21. B4-28. B4-29. B4-30. B4-32. B4-33. B4-40. B4-43. B4-45. B4-46. B4-47. B4-49. B4-50. B4-52 | 21 | 42.00 | IX |
| XVIII | B4-3. B4-26. B4-38 | 3 | 6.00 | VIII |
| XV | B4-4. B4-12 | 2 | 4.00 |  |
| LIII | B4-5 | 1 | 2.00 |  |
| XIII | B4-7. B4-15 | 2 | 4.00 | XI |
| LIV | B4-9. B4-27. B4-31 | 3 | 6.00 |  |
| LV | B4-11. B4-36 | 2 | 4.00 |  |
| LVI | B4-17 | 1 | 2.00 |  |
| VII | B4-18 | 1 | 2.00 | XXII |
| LVII | B4-20 | 1 | 2.00 |  |
| LVIII | B4-22 | 1 | 2.00 |  |
| XIX | B4-23 | 1 | 2.00 |  |
| LIX | B4-24 | 1 | 2.00 |  |
| XI | B4-25 | 1 | 2.00 | LIX |
| IV | B4-34 | 1 | 2.00 | XIII |
| LX | B4-35 | 1 | 2.00 |  |
| LXI | B4-37 | 1 | 2.00 |  |
| XXI | B4-39 | 1 | 2.00 |  |
| LXII | B4-42 | 1 | 2.00 |  |
| LXIII | B4-44 | 1 | 2.00 |  |
| LXIV | B4-48 | 1 | 2.00 |  |
| XVII | B4-51 | 1 | 2.00 |  |

| RFLP 2003 | B5 Isolates | Number of isolates | % in sample B5 | RFLP 2002 |
| --- | --- | --- | --- | --- |
| LXV | B5-1 | 1 | 1.92 |  |
| I | B5-2. B5-3. B5-4. B5-5. B5-6. B5-7. B5-8. B5-9. B5-10. B5-11. B5-12. B5-13. B5-14. B5-15. B5-16. B5-17. B5-18. B5-19. B5-20. B5-23. B5-24. B5-25. B5-27. B5-28. B5-29. B5-30. B5-31. B5-32. B5-34. B5-35. B5-36. B5-37. B5-38. B5-39. B5-42. B5-43. B5-44. B5-45. B5-46. B5-48. B5-49. B5-50. B5-51 | 43 | 82.69 | XXIII |
| LXVI | B5-21 | 1 | 1.92 |  |
| VI | B5-22. B5-26. B5-33 | 3 | 5.77 | LXVI |
| LXVII | B5-40 | 1 | 1.92 |  |
| III | B5-41. B5-52 | 2 | 3.85 |  |
| VII | B5-47 | 1 | 1.92 | XXII |

| RFLP 2003 | B6 Isolates | Number of isolates | % in sample B6 | RFLP 2002 |
| --- | --- | --- | --- | --- |
| LXVIII | B6-1 | 1 | 1.96 |  |
| LXIX | B6-2 | 1 | 1.96 |  |
| I | B6-3. B6-8. B6-12. B6-18. B6-20. B6-21. B6-25. B6-28. B6-31. B6-36. B6-38. B6-41. B6-43. B6-44. B6-45. B6-51 | 16 | 31.37 | XXIII |
| XXVI | B6-4. B6-37. B6-42 | 3 | 5.88 |  |
| XI | B6-5 | 1 | 1.96 | LIX |
| IV | B6-6. B6-17. B6-19. B6-32. B6-33. B6-39. B6-46. B6-47. B6-48 | 9 | 17.65 | XIII |
| XIII | B6-7. B6-50 | 2 | 3.92 | XI |
| VI | B6-9. B6-40 | 2 | 3.92 | LXVI |
| X | B6-10. B6-49 | 2 | 3.92 |  |
| XVIII | B6-11 | 1 | 1.96 | VIII |
| V | B6-13. B6-14. B6-24. B6-26 | 4 | 7.84 | IX |
| XXI | B6-15 | 1 | 1.96 |  |
| VII | B6-16 | 1 | 1.96 | XXII |
| LXX | B6-22 | 1 | 1.96 |  |
| LXII | B6-23 | 1 | 1.96 |  |
| XVII | B6-27 | 1 | 1.96 |  |
| III | B6-29 | 1 | 1.96 |  |
| XXXIV | B6-30 | 1 | 1.96 |  |
| XLIV | B6-34 | 1 | 1.96 |  |
| LXXI | B6-52 | 1 | 1.96 |  |

| RFLP 2003 | D2 Isolates | Number of isolates | % in sample D2 | RFLP 2002 |
| --- | --- | --- | --- | --- |
| LXXII | D2-1 | 1 | 1.92 |  |
| XI | D2-2 | 1 | 1.92 | LIX |
| LXXIII | D2-3 | 1 | 1.92 |  |
| LXXIV | D2-4. D2-5. D2-6. D2-11. D2-13. D2-14. D2-16. D2-17. D2-20. D2-22. D2-23. D2-24.  D2-26. D2-27. D2-28. D2-29. D2-30. D2-32. D2-33. D2-34. D2-35. D2-37. D2-39. D2-41. D2-44. D2-46. D2-47. D2-48. D2-50. D2-51. D2-52 | 31 | 59.62 |  |
| LXXV | D2-7 | 1 | 1.92 |  |
| LXXVI | D2-8. D2-38 | 2 | 3.85 |  |
| LXXVII | D2-9 | 1 | 1.92 |  |
| LXXVIII | D2-10 | 1 | 1.92 |  |
| LXXIX | D2-12 | 1 | 1.92 |  |
| LXXX | D2-15 | 1 | 1.92 |  |
| LXXXI | D2-18. D2-42 | 2 | 3.85 |  |
| LXXXII | D2-19. D2-31. D2-43 | 3 | 5.77 |  |
| LXXXIII | D2-21. D2-45 | 2 | 3.85 |  |
| LXXXIV | D2-25. D2-49 | 2 | 3.85 |  |
| V | D2-36 | 1 | 1.92 | IX |
| LXXXV | D2-40 | 1 | 1.92 |  |

| RFLP 2003 | D3 Isolates | Number of isolates | % in sample D3 | RFLP 2002 |
| --- | --- | --- | --- | --- |
| CXXII | D3-1. D3-5. D3-10. D3-11. D3-14. D3-15. D3-18. D3-20. D3-27. D3-31. D3-36. D3-38 | 12 | 23.53 |  |
| II | D3-2. D3-8. D3-28. D3-46 | 4 | 7.84 | VII |
| LXXIV | D3-3. D3-12. D3-34 | 3 | 5.88 |  |
| CXXIII | D3-4 | 1 | 1.96 |  |
| LXXVI | D3-6. D3-24 | 2 | 3.92 |  |
| V | D3-7. D3-16. D3-19. D3-29. D3-40 | 5 | 9.8 | IX |
| XXVI | D3-9 | 1 | 1.96 |  |
| LXXIX | D3-17. D3-37. D3-39. D3-51 | 4 | 7.84 |  |
| CXXIV | D3-21 | 1 | 1.96 |  |
| CXXV | D3-22 | 1 | 1.96 |  |
| CXXVI | D3-23 | 1 | 1.96 |  |
| XVIII | D3-25 | 1 | 1.96 | VIII |
| XIII | D3-26. D3-32 | 2 | 3.92 | XI |
| CXXVII | D3-30. D3-44. D3-49. D3-50. D3-52 | 5 | 9.8 |  |
| CXXVIII | D3-33 | 1 | 1.96 |  |
| CXXIX | D3-35 | 1 | 1.96 |  |
| CXXX | D3-41 | 1 | 1.96 |  |
| CXXXI | D3-42 | 1 | 1.96 |  |
| CXXXII | D3-43 | 1 | 1.96 |  |
| CXXXIII | D3-45 | 1 | 1.96 |  |
| LXXXII | D3-47 | 1 | 1.96 |  |
| CXXXIV | D3-48 | 1 | 1.96 |  |

| RFLP 2003 | E1 Isolates | Number of isolates | % in sample E1 | RFLP 2002 |
| --- | --- | --- | --- | --- |
| LXXXVI | E1-1 | 1 | 1.92 | XXVII |
| VII | E1-2. E1-9 | 2 | 3.85 | XXII |
| I | E1-3. E1-5. E1-8. E1-11. E1-16. E1-31. E1-33. E1-41. E1-43. E1-48. E1-49 | 11 | 21.15 | XXIII |
| II | E1-4. E1-10. E1-12. E1-26. E1-34. E1-37 | 6 | 11.54 | VII |
| V | E1-6. E1-30. E1-32 | 3 | 5.77 | IX |
| LXXXVII | E1-7. E1-28. E1-36 | 3 | 5.77 |  |
| LXXXVIII | E1-13 | 1 | 1.92 |  |
| IV | E1-14. E1-17. E1-19. E1-27. E1-29. E1-47 | 6 | 11.54 | XIII |
| LXXXIX | E1-15 | 1 | 1.92 |  |
| XC | E1-18 | 1 | 1.92 |  |
| XIII | E1-20. E1-35. E1-38. E1-42. E1-45. E1-50. E1-52 | 7 | 13.46 | XI |
| XCI | E1-21 | 1 | 1.92 |  |
| VI | E1-22. E1-25 | 2 | 3.85 | LXVI |
| XCII | E1-23 | 1 | 1.92 |  |
| XCIII | E1-24 | 1 | 1.92 |  |
| XCIV | E1-39 | 1 | 1.92 |  |
| XCV | E1-40. E1-51 | 2 | 3.85 |  |
| XCVI | E1-44 | 1 | 1.92 |  |
| XVIII | E1-46 | 1 | 1.92 | VIII |

| RFLP 2003 | F1 Isolates | Number of isolates | % in sample F1 | RFLP 2002 |
| --- | --- | --- | --- | --- |
| I | F1-1. F1-10. F1-27. F1-34 | 4 | 7.69 | XXIII |
| LXXXVI | F1-2. F1-7. F1-25. F1-33. F1-48 | 5 | 9.62 | XXVII |
| XCVII | F1-3. F1-14. F1-16. F1-47 | 4 | 7.6 | LXXIV |
| IV | F1-4. F1-18. F1-19. F1-20. F1-21. F1-29. F1-31. F1-43 | 8 | 15.38 | XIII |
| XI | F1-5. F1-17. F1-36 | 3 | 5.77 | LIX |
| VII | F1-6. F1-37. F1-38 | 3 | 5.77 | XXII |
| V | F1-8. F1-24. F1-30. F1-39. F1-40. F1-44 | 6 | 11.54 | IX |
| XXVI | F1-9. | 1 | 1.92 |  |
| XVIII | F1-11. F1-15. F1-49 | 3 | 5.77 | VIII |
| XCVIII | F1-12 | 1 | 1.92 |  |
| IC | F1-13 | 1 | 1.92 |  |
| II | F1-22 | 1 | 1.92 | VII |
| C | F1-23 | 1 | 1.92 |  |
| CI | F1-26 | 1 | 1.92 |  |
| XIII | F1-28. F1-35 | 2 | 3.85 | XI |
| CII | F1-32 | 1 | 1.92 |  |
| CIII | F1-41 | 1 | 1.92 |  |
| CIV | F1-42 | 1 | 1.92 | XXVIII |
| LIV | F1-45 | 1 | 1.92 |  |
| CV | F1-46 | 1 | 1.92 |  |
| CVI | F1-50. F1-51 | 2 | 3.85 |  |
| XV | F1-52 | 1 | 1.92 |  |

| RFLP 2003 | G1 Isolates | Number of isolates | % in sample G1 | RFLP 2002 |
| --- | --- | --- | --- | --- |
| CVII | G1-1 | 1 | 1.92 |  |
| XVIII | G1-2. G1-19. G1-32. G1-37. G1-45 | 5 | 9.62 | VIII |
| V | G1-3. G1-30. G1-51. G1-52 | 4 | 7.69 | IX |
| CVI | G1-4. G1-23. G1-38. G1-48 | 4 | 7.69 |  |
| CVIII | G1-5 | 1 | 1.92 |  |
| IL | G1-6 | 1 | 1.92 |  |
| CIX | G1-7. G1-15 | 2 | 3.85 |  |
| CIV | G1-8 | 1 | 1.92 | XXVIII |
| CX | G1-9. G1-10 | 2 | 3.85 |  |
| CXI | G1-11 | 1 | 1.92 |  |
| CXII | G1-12. G1-13 | 2 | 3.85 |  |
| CXIII | G1-14. G1-36 | 2 | 3.85 |  |
| CXIV | G1-16. G1-43 | 2 | 3.85 |  |
| XIII | G1-17. G1-20. G1-21. G1-42. G1-44. G1-47 | 6 | 11.54 | XI |
| CXV | G1-18. G1-35. G1-39 | 3 | 5.77 |  |
| XCV | G1-22 | 1 | 1.92 |  |
| IV | G1-24. G1-50 | 2 | 3.85 | XIII |
| CXVI | G1-25 | 1 | 1.92 |  |
| CXVII | G1-26 | 1 | 1.92 |  |
| CXVIII | G1-27. G1-46 | 2 | 3.85 |  |
| XI | G1-28. G1-29 | 2 | 3.85 | LIX |
| CXIX | G1-31 | 1 | 1.92 |  |
| LXXXVI | G1-33. G1-34 | 2 | 3.85 | XXVII |
| CXX | G1-40 | 1 | 1.92 |  |
| II | G1-41 | 1 | 1.92 | VII |
| CXXI | G1-49 | 1 | 1.92 |  |
