# Supplementary material for: Biodiversity of Indigenous Saccharomyces Populations from Old Wineries of South-Eastern Sicily (Italy): Preservation and Economic Potential
Source: PLoS One. 2012 Feb 29;7(2):e30428. doi: 10.1371/journal.pone.0030428 (PMC3290603; doi:10.1371/journal.pone.0030428)
Supplement: Table S2 — Analysis of the 2002 vintage. For each of the different strains (I to LXXXIX), the table shows the number of isolates for each of the samplings (A1, A2, A3, A4, B2, B3, D1), the total number of isolates per strain and the percentages of that strain in the population. (DOC) [file pone.0030428.s006.doc]

| Strains 2002 | Number of isolates per sample | | | | | | | Number of isolates in 2002 | % in 2002 |
| --- | --- | --- | --- | --- | --- | --- | --- | --- | --- |
|  | A1 | A2 | A3 | A4 | B2 | B3 | D1 |  |  |
| I | 5 | 1 |  |  | 1 |  | 1 | 8 | 2.27 |
| II | 2 |  |  |  |  |  |  | 2 | 0.57 |
| III | 1 |  |  |  |  |  |  | 1 | 0.28 |
| IV | 1 |  |  |  |  |  |  | 1 | 0.28 |
| V | 2 |  |  |  |  |  |  | 2 | 0.57 |
| VI | 1 |  |  |  |  |  |  | 1 | 0.28 |
| VII | 2 | 3 | 1 |  |  |  |  | 6 | 1.70 |
| VIII | 3 | 8 | 3 |  |  | 2 |  | 16 | 4.55 |
| IX | 4 | 8 | 2 | 1 | 6 | 6 |  | 27 | 7.67 |
| X | 1 |  |  |  |  |  |  | 1 | 0.28 |
| XI | 3 | 2 | 3 | 2 | 3 | 2 |  | 15 | 4.26 |
| XII | 1 |  |  |  |  |  |  | 1 | 0.28 |
| XIII | 2 | 6 | 12 | 5 | 13 | 5 | 1 | 44 | 12.50 |
| XIV | 1 |  |  |  |  |  |  | 1 | 0.28 |
| XV | 1 | 2 |  |  | 1 |  | 1 | 5 | 1.42 |
| XVI | 1 |  |  |  |  |  |  | 1 | 0.28 |
| XVII | 1 |  | 1 | 7 |  |  |  | 9 | 2.56 |
| XVIII | 1 |  |  |  |  |  |  | 1 | 0.28 |
| XIX | 3 |  |  |  |  |  |  | 3 | 0.85 |
| XX | 1 |  | 1 | 1 | 2 | 1 |  | 6 | 1.70 |
| XXI | 1 | 2 |  |  |  |  | 1 | 4 | 1.14 |
| XXII | 4 |  | 1 | 1 |  | 1 |  | 7 | 1.99 |
| XXIII | 2 |  | 13 | 22 | 4 | 9 |  | 50 | 14.20 |
| XXIV | 1 |  |  |  |  |  |  | 1 | 0.28 |
| XXV | 1 |  |  |  | 1 |  |  | 2 | 0.57 |
| XXVI | 1 |  |  |  |  |  |  | 1 | 0.28 |
| XXVII | 2 |  |  |  |  |  |  | 2 | 0.57 |
| XXVIII | 1 |  |  |  |  |  |  | 1 | 0.28 |
| XXIX | 1 |  |  |  |  |  |  | 1 | 0.28 |
| XXX | 1 |  |  |  |  |  |  | 1 | 0.28 |
| XXXI |  | 1 |  |  |  |  |  | 1 | 0.28 |
| XXXII |  | 2 |  |  |  |  |  | 2 | 0.57 |
| XXXIII |  | 1 |  |  |  |  |  | 1 | 0.28 |
| XXXIV |  | 2 |  |  |  |  |  | 2 | 0.57 |
| XXXV |  | 1 |  |  |  |  |  | 1 | 0.28 |
| XXXVI |  | 1 |  |  |  |  |  | 1 | 0.28 |
| XXXVII |  | 1 |  |  |  |  |  | 1 | 0.28 |
| XXXVIII |  | 1 |  |  |  |  |  | 1 | 0.28 |
| XXXIX |  | 1 |  |  |  |  |  | 1 | 0.28 |
| XL |  | 1 |  |  |  |  |  | 1 | 0.28 |
| XLI |  | 1 |  |  |  |  | 37 | 38 | 10.80 |
| XLII |  | 1 |  |  |  |  |  | 1 | 0.28 |
| XLIII |  | 1 |  | 1 | 1 |  |  | 3 | 0.85 |
| XLIV |  | 1 |  |  | 1 |  |  | 2 | 0.57 |
| XLV |  | 1 |  |  |  |  |  | 1 | 0.28 |
| XLVI |  | 1 |  |  |  |  |  | 1 | 0.28 |
| XLVII |  |  | 1 |  |  | 1 |  | 2 | 0.57 |
| XLVIII |  |  | 1 |  |  |  |  | 1 | 0.28 |
| IL |  |  | 2 |  |  |  |  | 2 | 0.57 |
| L |  |  | 2 | 2 |  |  |  | 4 | 1.14 |
| LI |  |  | 1 |  |  |  |  | 1 | 0.28 |
| LII |  |  | 1 |  |  |  |  | 1 | 0.28 |
| LIII |  |  | 1 |  |  |  |  | 1 | 0.28 |
| LIV |  |  | 1 |  |  |  |  | 1 | 0.28 |
| LV |  |  | 1 |  |  |  |  | 1 | 0.28 |
| LVI |  |  | 2 |  |  |  |  | 2 | 0.57 |
| LVII |  |  | 1 |  |  | 1 |  | 2 | 0.57 |
| LVIII |  |  |  | 1 |  |  |  | 1 | 0.28 |
| LIX |  |  |  | 3 | 4 | 8 | 1 | 16 | 4.55 |
| LX |  |  |  | 1 |  |  |  | 1 | 0.28 |
| LXI |  |  |  | 1 |  |  |  | 1 | 0.28 |
| LXII |  |  |  | 1 |  |  |  | 1 | 0.28 |
| LXIII |  |  |  | 1 |  |  |  | 1 | 0.28 |
| LXIV |  |  |  |  | 1 |  |  | 1 | 0.28 |
| LXV |  |  |  |  | 1 |  |  | 1 | 0.28 |
| LXVI |  |  |  |  | 3 |  |  | 3 | 0.85 |
| LXVII |  |  |  |  | 1 |  |  | 1 | 0.28 |
| LXVIII |  |  |  |  | 3 |  |  | 3 | 0.85 |
| LXIX |  |  |  |  | 1 | 1 |  | 2 | 0.57 |
| LXX |  |  |  |  | 1 |  |  | 1 | 0.28 |
| LXXI |  |  |  |  | 1 |  |  | 1 | 0.28 |
| LXXII |  |  |  |  | 1 |  |  | 1 | 0.28 |
| LXXIII |  |  |  |  |  | 2 |  | 2 | 0.57 |
| LXXIV |  |  |  |  |  | 2 |  | 2 | 0.57 |
| LXXV |  |  |  |  |  | 1 |  | 1 | 0.28 |
| LXXVI |  |  |  |  |  | 1 |  | 1 | 0.28 |
| LXXVII |  |  |  |  |  | 1 |  | 1 | 0.28 |
| LXXVIII |  |  |  |  |  | 1 |  | 1 | 0.28 |
| LXXIX |  |  |  |  |  | 1 |  | 1 | 0.28 |
| LXXX |  |  |  |  |  | 1 |  | 1 | 0.28 |
| LXXXI |  |  |  |  |  | 1 |  | 1 | 0.28 |
| LXXXII |  |  |  |  |  | 1 |  | 1 | 0.28 |
| LXXXIII |  |  |  |  |  | 1 |  | 1 | 0.28 |
| LXXXIV |  |  |  |  |  |  | 1 | 1 | 0.28 |
| LXXXV |  |  |  |  |  |  | 2 | 2 | 0.57 |
| LXXXVI |  |  |  |  |  |  | 1 | 1 | 0.28 |
| LXXXVII |  |  |  |  |  |  | 1 | 1 | 0.28 |
| LXXXVIII |  |  |  |  |  |  | 1 | 1 | 0.28 |
| LXXXIX |  |  |  |  |  |  | 1 | 1 | 0.28 |
| total | 52 | 50 | 51 | 50 | 50 | 50 | 49 | 352 | 100.00 |
