# Supplementary material for: Biodiversity of Indigenous Saccharomyces Populations from Old Wineries of South-Eastern Sicily (Italy): Preservation and Economic Potential
Source: PLoS One. 2012 Feb 29;7(2):e30428. doi: 10.1371/journal.pone.0030428 (PMC3290603; doi:10.1371/journal.pone.0030428)
Supplement: Table S3 — Analysis of the 2003 vintage. For each of the different strains (I to CXXXIV), the table shows the corresponding 2002 strain, the number of isolates for each of the samplings (A5, A6, A7, B4, B5, B6, D2, D3, E1, F1, G), the total number of isolates per strain and the percentages of that strain in the population. (DOC) [file pone.0030428.s007.doc]

| Strains 2003 | Strains 2002 | Number of isolates per sample | | | | | | | | | | | Number of isolates in 2003 | % in 2003 |
| --- | --- | --- | --- | --- | --- | --- | --- | --- | --- | --- | --- | --- | --- | --- |
|  |  | A5 | A6 | A7 | B4 | B5 | B6 | D2 | D3 | E1 | F1 | G1 |  |  |
| I | XXIII | 6 | 2 | 6 |  | 43 | 16 |  |  | 11 | 4 |  | 88 | 15.55 |
| II | VII | 2 |  | 2 |  |  |  |  | 4 | 6 | 1 | 1 | 16 | 2.83 |
| III |  | 2 |  |  |  | 2 | 1 |  |  |  |  |  | 5 | 0.88 |
| IV | XIII | 9 | 15 | 12 | 1 |  | 9 |  |  | 6 | 8 | 2 | 62 | 10.95 |
| V | IX | 5 | 1 | 1 | 21 |  | 4 | 1 | 5 | 3 | 6 | 4 | 51 | 9.01 |
| VI | LXVI | 3 |  |  |  | 3 | 2 |  |  | 2 |  |  | 10 | 1.77 |
| VII | XXII | 1 | 5 | 4 | 1 | 1 | 1 |  |  | 2 | 3 |  | 18 | 3.18 |
| VIII |  | 1 |  |  |  |  |  |  |  |  |  |  | 1 | 0.18 |
| IX |  | 1 |  |  |  |  |  |  |  |  |  |  | 1 | 0.18 |
| X |  | 1 |  |  |  |  | 2 |  |  |  |  |  | 3 | 0.53 |
| XI | LIX | 1 | 1 | 1 | 1 |  | 1 | 1 |  |  | 3 | 2 | 11 | 1.94 |
| XII |  | 1 |  |  |  |  |  |  |  |  |  |  | 1 | 0.18 |
| XIII | XI | 3 | 6 | 2 | 2 |  | 2 |  | 2 | 7 | 2 | 6 | 32 | 5.65 |
| XIV |  | 1 |  |  |  |  |  |  |  |  |  |  | 1 | 0.18 |
| XV |  | 1 | 1 | 2 | 2 |  |  |  |  |  | 1 |  | 7 | 1.24 |
| XVI |  | 1 |  |  |  |  |  |  |  |  |  |  | 1 | 0.18 |
| XVII |  | 1 |  |  | 1 |  | 1 |  |  |  |  |  | 3 | 0.53 |
| XVIII | VIII | 1 | 4 | 5 | 3 |  | 1 |  | 1 | 1 | 3 | 5 | 24 | 4.24 |
| XIX |  | 2 | 1 | 1 | 1 |  |  |  |  |  |  |  | 5 | 0.88 |
| XX |  | 1 | 2 |  |  |  |  |  |  |  |  |  | 3 | 0.53 |
| XXI |  | 1 |  |  | 1 |  | 1 |  |  |  |  |  | 3 | 0.53 |
| XXII |  | 1 |  |  |  |  |  |  |  |  |  |  | 1 | 0.18 |
| XXIII |  | 1 |  |  |  |  |  |  |  |  |  |  | 1 | 0.18 |
| XXIV |  | 1 |  |  |  |  |  |  |  |  |  |  | 1 | 0.18 |
| XXV |  | 1 |  |  |  |  |  |  |  |  |  |  | 1 | 0.18 |
| XXVI |  | 1 |  |  |  |  | 3 |  | 1 |  | 1 |  | 6 | 1.06 |
| XXVII |  | 1 |  |  |  |  |  |  |  |  |  |  | 1 | 0.18 |
| XXVIII |  |  | 1 |  |  |  |  |  |  |  |  |  | 1 | 0.18 |
| XXIX |  |  | 1 |  |  |  |  |  |  |  |  |  | 1 | 0.18 |
| XXX |  |  | 1 |  |  |  |  |  |  |  |  |  | 1 | 0.18 |
| XXXI |  |  | 2 | 1 |  |  |  |  |  |  |  |  | 3 | 0.53 |
| XXXII |  |  | 2 |  |  |  |  |  |  |  |  |  | 2 | 0.35 |
| XXXIII |  |  | 1 |  |  |  |  |  |  |  |  |  | 1 | 0.18 |
| XXXIV |  |  | 1 |  |  |  | 1 |  |  |  |  |  | 2 | 0.35 |
| XXXV |  |  | 1 |  |  |  |  |  |  |  |  |  | 1 | 0.18 |
| XXXVI |  |  | 1 |  |  |  |  |  |  |  |  |  | 1 | 0.18 |
| XXXVII |  |  | 1 |  |  |  |  |  |  |  |  |  | 1 | 0.18 |
| XXXVIII |  |  | 1 |  |  |  |  |  |  |  |  |  | 1 | 0.18 |
| XL |  |  | 1 |  |  |  |  |  |  |  |  |  | 1 | 0.18 |
| XLII |  |  |  | 1 |  |  |  |  |  |  |  |  | 1 | 0.18 |
| XLIII |  |  |  | 4 |  |  |  |  |  |  |  |  | 4 | 0.71 |
| XLIV |  |  |  | 1 |  |  | 1 |  |  |  |  |  | 2 | 0.35 |
| XLV |  |  |  | 1 |  |  |  |  |  |  |  |  | 1 | 0.18 |
| XLVI |  |  |  | 1 |  |  |  |  |  |  |  |  | 1 | 0.18 |
| XLVII |  |  |  | 2 |  |  |  |  |  |  |  |  | 2 | 0.35 |
| XLVIII |  |  |  | 1 |  |  |  |  |  |  |  |  | 1 | 0.18 |
| IL |  |  |  | 1 |  |  |  |  |  |  |  | 1 | 2 | 0.35 |
| L |  |  |  | 1 |  |  |  |  |  |  |  |  | 1 | 0.18 |
| LI |  |  |  | 1 |  |  |  |  |  |  |  |  | 1 | 0.18 |
| LII |  |  |  |  | 1 |  |  |  |  |  |  |  | 1 | 0.18 |
| LIII |  |  |  |  | 1 |  |  |  |  |  |  |  | 1 | 0.18 |
| LIV |  |  |  |  | 3 |  |  |  |  |  | 1 |  | 4 | 0.71 |
| LV |  |  |  |  | 2 |  |  |  |  |  |  |  | 2 | 0.35 |
| LVI |  |  |  |  | 1 |  |  |  |  |  |  |  | 1 | 0.18 |
| LVII |  |  |  |  | 1 |  |  |  |  |  |  |  | 1 | 0.18 |
| LVIII |  |  |  |  | 1 |  |  |  |  |  |  |  | 1 | 0.18 |
| LIX |  |  |  |  | 1 |  |  |  |  |  |  |  | 1 | 0.18 |
| LX |  |  |  |  | 1 |  |  |  |  |  |  |  | 1 | 0.18 |
| LXI |  |  |  |  | 1 |  |  |  |  |  |  |  | 1 | 0.18 |
| LXII |  |  |  |  | 1 |  | 1 |  |  |  |  |  | 2 | 0.35 |
| LXIII |  |  |  |  | 1 |  |  |  |  |  |  |  | 1 | 0.18 |
| LXIV |  |  |  |  | 1 |  |  |  |  |  |  |  | 1 | 0.18 |
| LXV |  |  |  |  |  | 1 |  |  |  |  |  |  | 1 | 0.18 |
| LXVI |  |  |  |  |  | 1 |  |  |  |  |  |  | 1 | 0.18 |
| LXVII |  |  |  |  |  | 1 |  |  |  |  |  |  | 1 | 0.18 |
| LXVIII |  |  |  |  |  |  | 1 |  |  |  |  |  | 1 | 0.18 |
| LXIX |  |  |  |  |  |  | 1 |  |  |  |  |  | 1 | 0.18 |
| LXX |  |  |  |  |  |  | 1 |  |  |  |  |  | 1 | 0.18 |
| LXXI |  |  |  |  |  |  | 1 |  |  |  |  |  | 1 | 0.18 |
| LXXII |  |  |  |  |  |  |  | 1 |  |  |  |  | 1 | 0.18 |
| LXXIII |  |  |  |  |  |  |  | 1 |  |  |  |  | 1 | 0.18 |
| LXXIV |  |  |  |  |  |  |  | 31 | 3 |  |  |  | 34 | 6.01 |
| LXXV |  |  |  |  |  |  |  | 1 |  |  |  |  | 1 | 0.18 |
| LXXVI |  |  |  |  |  |  |  | 2 | 2 |  |  |  | 4 | 0.71 |
| LXXVII |  |  |  |  |  |  |  | 1 |  |  |  |  | 1 | 0.18 |
| LXXVIII |  |  |  |  |  |  |  | 1 |  |  |  |  | 1 | 0.18 |
| LXXIX |  |  |  |  |  |  |  | 1 | 4 |  |  |  | 5 | 0.88 |
| LXXX |  |  |  |  |  |  |  | 1 |  |  |  |  | 1 | 0.18 |
| LXXXI |  |  |  |  |  |  |  | 2 |  |  |  |  | 2 | 0.35 |
| LXXXII |  |  |  |  |  |  |  | 3 | 1 |  |  |  | 4 | 0.71 |
| LXXXIII |  |  |  |  |  |  |  | 2 |  |  |  |  | 2 | 0.35 |
| LXXXIV |  |  |  |  |  |  |  | 2 |  |  |  |  | 2 | 0.35 |
| LXXXV |  |  |  |  |  |  |  | 1 |  |  |  |  | 1 | 0.18 |
| LXXXVI | XXVII |  |  |  |  |  |  |  |  | 1 | 5 | 2 | 8 | 1.41 |
| LXXXVII |  |  |  |  |  |  |  |  |  | 3 |  |  | 3 | 0.53 |
| LXXXVIII |  |  |  |  |  |  |  |  |  | 1 |  |  | 1 | 0.18 |
| LXXXIX |  |  |  |  |  |  |  |  |  | 1 |  |  | 1 | 0.18 |
| XC |  |  |  |  |  |  |  |  |  | 1 |  |  | 1 | 0.18 |
| XCI |  |  |  |  |  |  |  |  |  | 1 |  |  | 1 | 0.18 |
| XCII |  |  |  |  |  |  |  |  |  | 1 |  |  | 1 | 0.18 |
| XCIII |  |  |  |  |  |  |  |  |  | 1 |  |  | 1 | 0.18 |
| XCIV |  |  |  |  |  |  |  |  |  | 1 |  |  | 1 | 0.18 |
| XCV |  |  |  |  |  |  |  |  |  | 2 |  | 1 | 3 | 0.53 |
| XCVI |  |  |  |  |  |  |  |  |  | 1 |  |  | 1 | 0.18 |
| XCVII | LXXIV |  |  |  |  |  |  |  |  |  | 4 |  | 4 | 0.71 |
| XCVIII |  |  |  |  |  |  |  |  |  |  | 1 |  | 1 | 0.18 |
| IC |  |  |  |  |  |  |  |  |  |  | 1 |  | 1 | 0.18 |
| C |  |  |  |  |  |  |  |  |  |  | 1 |  | 1 | 0.18 |
| CI |  |  |  |  |  |  |  |  |  |  | 1 |  | 1 | 0.18 |
| CII |  |  |  |  |  |  |  |  |  |  | 1 |  | 1 | 0.18 |
| CIII |  |  |  |  |  |  |  |  |  |  | 1 |  | 1 | 0.18 |
| CIV | XXVIII |  |  |  |  |  |  |  |  |  | 1 | 1 | 2 | 0.35 |
| CV |  |  |  |  |  |  |  |  |  |  | 1 |  | 1 | 0.18 |
| CVI |  |  |  |  |  |  |  |  |  |  | 2 | 4 | 6 | 1.06 |
| CVII |  |  |  |  |  |  |  |  |  |  |  | 1 | 1 | 0.18 |
| CVIII |  |  |  |  |  |  |  |  |  |  |  | 1 | 1 | 0.18 |
| CIX |  |  |  |  |  |  |  |  |  |  |  | 2 | 2 | 0.35 |
| CX |  |  |  |  |  |  |  |  |  |  |  | 2 | 2 | 0.35 |
| CXI |  |  |  |  |  |  |  |  |  |  |  | 1 | 1 | 0.18 |
| CXII |  |  |  |  |  |  |  |  |  |  |  | 2 | 2 | 0.35 |
| CXIII |  |  |  |  |  |  |  |  |  |  |  | 2 | 2 | 0.35 |
| CXIV |  |  |  |  |  |  |  |  |  |  |  | 2 | 2 | 0.35 |
| CXV |  |  |  |  |  |  |  |  |  |  |  | 3 | 3 | 0.53 |
| CXVI |  |  |  |  |  |  |  |  |  |  |  | 1 | 1 | 0.18 |
| CXVII |  |  |  |  |  |  |  |  |  |  |  | 1 | 1 | 0.18 |
| CXVIII |  |  |  |  |  |  |  |  |  |  |  | 2 | 2 | 0.35 |
| CXIX |  |  |  |  |  |  |  |  |  |  |  | 1 | 1 | 0.18 |
| CXX |  |  |  |  |  |  |  |  |  |  |  | 1 | 1 | 0.18 |
| CXXI |  |  |  |  |  |  |  |  |  |  |  | 1 | 1 | 0.18 |
| CXXII |  |  |  |  |  |  |  |  | 12 |  |  |  | 12 | 2.12 |
| CXXIII |  |  |  |  |  |  |  |  | 1 |  |  |  | 1 | 0.18 |
| CXXIV |  |  |  |  |  |  |  |  | 1 |  |  |  | 1 | 0.18 |
| CXXV |  |  |  |  |  |  |  |  | 1 |  |  |  | 1 | 0.18 |
| CXXVI |  |  |  |  |  |  |  |  | 1 |  |  |  | 1 | 0.18 |
| CXXVII |  |  |  |  |  |  |  |  | 5 |  |  |  | 5 | 0.88 |
| CXXVIII |  |  |  |  |  |  |  |  | 1 |  |  |  | 1 | 0.18 |
| CXXIX |  |  |  |  |  |  |  |  | 1 |  |  |  | 1 | 0.18 |
| CXXX |  |  |  |  |  |  |  |  | 1 |  |  |  | 1 | 0.18 |
| CXXXI |  |  |  |  |  |  |  |  | 1 |  |  |  | 1 | 0.18 |
| CXXXII |  |  |  |  |  |  |  |  | 1 |  |  |  | 1 | 0.18 |
| CXXXIII |  |  |  |  |  |  |  |  | 1 |  |  |  | 1 | 0.18 |
| CXXXIV |  |  |  |  |  |  |  |  | 1 |  |  |  | 1 | 0.18 |
| total |  | 51 | 52 | 51 | 50 | 52 | 51 | 52 | 51 | 52 | 52 | 52 | 566 | 100.00 |
