# Supplementary material for: Biodiversity of Indigenous Saccharomyces Populations from Old Wineries of South-Eastern Sicily (Italy): Preservation and Economic Potential
Source: PLoS One. 2012 Feb 29;7(2):e30428. doi: 10.1371/journal.pone.0030428 (PMC3290603; doi:10.1371/journal.pone.0030428)
Supplement: Table S4 — Delta sequence amplification pattern analyses. All the isolates belonging to the same strain (indicated in the first two columns) are grouped according to their different delta sequence amplification patterns. Note that the groups indicated from 1 to 19 are not the same from one row to the next since the strains are different (e.g. group 1 for class I is different from group 1 for class II). (DOC) [file pone.0030428.s008.doc]

| Strains 2002 | Strains 2003 | Results | **1** | **2** | **3** | **4** | **5** | **6** | **7** | **8** | **9** | **10** | **11** | **12** | **13** | **14** | **15** | **16** | **17** | **18** | **19** |
| --- | --- | --- | --- | --- | --- | --- | --- | --- | --- | --- | --- | --- | --- | --- | --- | --- | --- | --- | --- | --- | --- |
| I |  | 8 isolates 5 delta patterns | A1-1, A1-17, A1-28 | B2-2, D1-43 | A1-31 | A1-50 | A2-12 |  |  |  |  |  |  |  |  |  |  |  |  |  |  |
| II |  | 2 isolates 1 delta pattern | A1-2, A1-6 |  |  |  |  |  |  |  |  |  |  |  |  |  |  |  |  |  |  |
| V |  | 2 isolates 1 delta pattern | A1-5, A1-33 |  |  |  |  |  |  |  |  |  |  |  |  |  |  |  |  |  |  |
| VII | II | 22 isolates 12 delta patterns | A2-11, A2-26, A3-51, G1-41 | E1-37, E1-4, E1-10 | A5-7, E1-12 | A1-8 | A1-44 | A2-23, A5-2 | A7-6, F1-22 | A7-47 | D3-2 | D3-8, D3-46 | E1-26, E1-34 | D3-28 |  |  |  |  |  |  |  |
| VIII | XVIII | 40 isolates 15 delta patterns | A6-21, A7-28, B3-5, B3-32, B4-3, B4-26, B4-38, B6-11, F1-11, F1-15, F1-49 | A1-9, A2-19, A2-44, A3-14 | A2-24, A2-30, A2-32, E1-46 | A1-36, A7-10 A7-13, A7-22 | A3-25, A3-48 | A6-39, G1-37, G1-45 | A1-47, A7-52 | A2-18, A2-51 | A2-53 | A5-29 | A6-29 | D3-25 | G1-19, G1-32 | A6-49 | G1-2 |  |  |  |  |
| IX | V | 78 isolates 19 delta patterns | B2-29, B2-36, B2-43, B3-34, B3-38, B4-30, B4-50, B4-52, B6-26, D3-7, D3-16, D3-19, D3-29, D3-40, E1-6, E1-30, F1-8, F1-24, F1-30, F1-39, F1-40, F1-44, G1-3 | A1-24, A5-14, A5-28, A5-36, B2-41, B3-13, B3-18, B3-24, B3-33, B4-2, B4-10, B4-13, B4-21, B4-33, B4-40, B4-43, B4-45, B4-47, B6-13, B6-14, G1-30, G1-51, G1-52 | A1-10, A1-22, A1-34, A2-22, A2-34, A2-39, A2-43, A2-46, A3-11, A3-37, A4-8 | A5-5 | A5-23 | A6-46 | B6-24 | A2-5 | A2-9 | A2-35 | B2-15 | E1-32 | B4-16, B4-19, B4 28, B4-29 | B4-32, B4-46, B4-49 | B4-8 | B4-14 | B2-23 | A7-33 | D2-36 |
| XI | XIII | 47 isolates 11 delta pattern | A2-48, A3-22, A3-35, A3-49, A4-26, A4-29, A5-30, A6-6, A6-26, A6-30, A6-48, A6-52, A7-32, B2-19 | G1-17, G1-21, G1-42, G1-44, G1-47, B6-50, D3-26, D3-32, E1-35, E1-38, E1- 42, E1-45, E1-50, E1-52 | A1-12, B3-43, B3-44, B4-7, | A7-50, B4-15B6-7, F1-35, G1-20 | E1-20 | A1-37, A1-48, B2-39, A6-43 | A2-52 | B2-14 | F1-28 | A5-21 | A5-45 |  |  |  |  |  |  |  |  |
| XIII | IV | 106 isolates, 18 delta patterns | A1-14, A1-25, A2-6, A2-10, A2-17, A2-21, A2-29, A3-1, A3-5, A3-9, A3-18, A3-20, A3-27, A3-36, A3-40, A3-42, A3-50, A4-5, A4-25, A4-36, A5-4, A5-24, A7-11, A7-30, A7-39, A7-40, A7-42 | A2-14, A3-7, A3-19, A5-31, A5-33, A5-52, A6-7, A6-12, A6-16, A7-14, A7-24, A7-44, B2-38, B3-8, B3-45 | A4-16, A5-39, A5-44, A6-1 A6-3, A6-4, A6-9, A6-15, B2-6, B2-20, B2-26, B2-30, B2-35, B2-49, B2-50, B3-16, B3-21 | A4-38, B3-40, B2-5, B2-9, B2-12, B2-17, B2-34, D1-1 | B4-34, B6-17, B6-19, B6-39, B6-46, B6-48, E1-27, E1-47, F1-4, F1-18, F1-21, F1-29, F1-31 | A6-34, A6-35, A6-38 | A6-40, A6-42, A6-44, A6-50 | A7-18, A7-21, A7-43 | B6-6, B6-47 | B6-32, B6-33 | E1-19, E1-29, F1-19, F1-20 | E1-14 | E1-17 | G1-50 | G1-24 | A5-19 | A5-42 | A7-12, F1-43 |  |
| XV |  | 5 isolates 4 delta patterns | A1-16, A2-37 | A2-3 | D1-2 | B2-40 |  |  |  |  |  |  |  |  |  |  |  |  |  |  |  |
| XVII |  | 9 isolates 3 delta patterns | A4-2, A4-6, A4-17, A4-24, A4-27, A4-32, A4-52 | A1-19 | A3-4 |  |  |  |  |  |  |  |  |  |  |  |  |  |  |  |  |
| XIX |  | 3 isolates 1 delta pattern | A1-21, A1-39, A1-51 |  |  |  |  |  |  |  |  |  |  |  |  |  |  |  |  |  |  |
| XX |  | 6 isolates 1 delta pattern | A1-23, A3-23, A4-34, B2-22, B2-28, B3-7 |  |  |  |  |  |  |  |  |  |  |  |  |  |  |  |  |  |  |
| XXI |  | 4 isolates 1 delta pattern | A1-26, A2-1, A2-36, D1-35 |  |  |  |  |  |  |  |  |  |  |  |  |  |  |  |  |  |  |
| XXII | VII | 25 isolates 7 delta patterns | A1-29, A1-30, A1-41, A3-6, A5-12, A6-14, A6-24, A6-25, A7-16, A7-29, A7-34, B3-49, B4-18, B5-47, E1-9, F1-6, F1-37 | A6-37, A6-45 | A1-27 | A7-15 | B6-16 | E1-2, F1-38 | A4-14 |  |  |  |  |  |  |  |  |  |  |  |  |
| XXIII | I | 138 isolates 9 delta patterns | A1-32, A1-49, A3-3, A3-10, A3-16, A3-21, A3-24, A3-28, A3-29,A3-31, A3-38, A3-41, A3-43, A3-44, A3-46, A5-1, A5-8, A5-9, A5-11, A5-17, A5-41, A7-1, A7-26, A7-31, A7-45, A7-48, B2-7, B2-16, B2-45, B2-47, B3-1, B3-2, B3-12, B3-15, B3-20, B3-26, B3-27, B3-29, B5-2, B5-3, B5-4, B5-5, B5-6, B5-7, B5-9, B5-10, B5-11, B5-12, B5-13, B5-14, B5-17, B5-18, B5-19, B5-20, B5-23, B5-25, B5-27, B5-28, B5-29, B5-30, B5-31, B5-32, B5-34, B5-35, B5-36, B5-37, B5-38, B5-39, B5-42, B5-43, B5-44, B5-45, B5-46, B5-48, B5-49, B5-50, B5-51, B6-3, B6-8, B6-12, B6-18, B6-20, B6-21, B6-25, B6-28, B6-36, B6-38, B6-41, B6-43, B6-44, B6-45, B6-51, E1-3, E1-5, E1-8, E1-11, E1-49, F1-1, F1-10, F1-27 | A4-4, A4-10, A4-28, A4-30, A4-33, A4-37, A4-39, A4-40, A4-43, A4-44, A4-46, A4-47, A4-51 | B5-8, B5-15, B5-16, E1-31, E1-33, E1-41, E1-43, E1-48 | A4-12, A4-13, A4-18, A4-19, A4-20, A4-21, A4-22, A4-23 | A6-28, A6-32, B3-31 | A7-25 | A4-11 | E1-16, F1-34 | B5-24, B6-31 |  |  |  |  |  |  |  |  |  |  |
| XXV |  | 2 isolates 2 delta patterns | A1-38 | B2-24 |  |  |  |  |  |  |  |  |  |  |  |  |  |  |  |  |  |
| XXVII | LXXXVI | 10 isolates7 delta patterns | F1-48, G1-33, G1-34 | A1-52, F1-2 | A1-42 | E1-1 | F1-7 | F1-25 | F1-33 |  |  |  |  |  |  |  |  |  |  |  |  |
| XXVIII | CIV | 3 isolates3 delta patterns | A1-43 | F1-42 | G1-8 |  |  |  |  |  |  |  |  |  |  |  |  |  |  |  |  |
| XXXII |  | 2 isolates2 delta patterns | A2-4 | A2-7 |  |  |  |  |  |  |  |  |  |  |  |  |  |  |  |  |  |
| XXXIV |  | 2 isolates2 delta patterns | A2-16 | A2-49 |  |  |  |  |  |  |  |  |  |  |  |  |  |  |  |  |  |
| XLI |  | 38 isolates10 delta patterns | D1-5, D1-7, D1-8, D1-10, D1-11, D1-12, D1-13, D1-16, D1-18, D1-22, D1-26, D1-27, D1-28, D1-29, D1-30, D1-32, D1-33, D1-38, D1-44, D1-48, D1-49 | D1-34, D1-46 | D1-6, D1-17 | D1-23, D1-31, D1-39, D1-41, D1-45 | A2-40, D1-50 | D1-37 | D1-20 | D1-47 | D1-9 | D1-24, D1-25 |  |  |  |  |  |  |  |  |  |
| XLIII |  | 3 isolates 2 delta patterns | A2-42, A4-9 | B2-46 |  |  |  |  |  |  |  |  |  |  |  |  |  |  |  |  |  |
| XLIV |  | 2 isolates 2 delta patterns | A2-45 | B2-48 |  |  |  |  |  |  |  |  |  |  |  |  |  |  |  |  |  |
| XLVII |  | 2 isolates 2 delta patterns | A3-2 | B3-14 |  |  |  |  |  |  |  |  |  |  |  |  |  |  |  |  |  |
| IL |  | 2 isolates 1 delta pattern | A3-12, A3-15 |  |  |  |  |  |  |  |  |  |  |  |  |  |  |  |  |  |  |
| L |  | 4 isolates 2 delta patterns | A3-30, A4-1, A4-3 | A3-13 |  |  |  |  |  |  |  |  |  |  |  |  |  |  |  |  |  |
| LVI |  | 2 isolates 2 delta patterns | A3-39 | A3-45 |  |  |  |  |  |  |  |  |  |  |  |  |  |  |  |  |  |
| LVII |  | 2 isolates 2 delta patterns | A3-47 | B3-37 |  |  |  |  |  |  |  |  |  |  |  |  |  |  |  |  |  |
| LIX | XI | 27 isolates 7 delta patterns | B2-3, B2-8, B2-18, B2-21, B3-9, B3-11, B3-19, B3-23, B3-35, B3-39, B3-46, B3-48, B4-25, B6-5, D2-2, F1-5, F1-36, G1-29 | A4-15, A4-35 | A4-48, A5-18, A7-17 | F1-17 | A6-51 | G1-28 | D1-14 |  |  |  |  |  |  |  |  |  |  |  |  |
| LXVI | VI | 13 isolates 5 delta patterns | B2-10, B2-13, B2-27, B5-22, B6-40, E1-22 | B5-26, B5-33, B6-9 | A5-10, A5-47 | A5-50 | E1-25 |  |  |  |  |  |  |  |  |  |  |  |  |  |  |
| LXVIII |  | 3 isolates 1 delta pattern | B2-25, B2-31, B2-32 |  |  |  |  |  |  |  |  |  |  |  |  |  |  |  |  |  |  |
| LXIX |  | 2 isolates 1 delta pattern | B2-33, B3-22 |  |  |  |  |  |  |  |  |  |  |  |  |  |  |  |  |  |  |
| LXXIII |  | 2 isolates1 delta pattern | B3-3, B3-25 |  |  |  |  |  |  |  |  |  |  |  |  |  |  |  |  |  |  |
| LXXIV | XCVII | 6 isolates1 delta pattern | B3-4, B3-28, F1-3, F1-14, F1-16, F1-47 |  |  |  |  |  |  |  |  |  |  |  |  |  |  |  |  |  |  |
| LXXXV |  | 2 isolates 2 delta patterns | D1-4 | D1-36 |  |  |  |  |  |  |  |  |  |  |  |  |  |  |  |  |  |
|  | III | 5 isolates 2 delta patterns | B5-41, B5-52, B6-29 | A5-3, A5-6 |  |  |  |  |  |  |  |  |  |  |  |  |  |  |  |  |  |
|  | X | 3 isolates 2 delta patterns | B6-10, B6-49 | A5-16 |  |  |  |  |  |  |  |  |  |  |  |  |  |  |  |  |  |
|  | XV | 7 isolates 3 delta patterns | B4-4, B4-12, F1-52 | A5-25, A7-8, A7-37 | A6-41 |  |  |  |  |  |  |  |  |  |  |  |  |  |  |  |  |
|  | XVII | 3 isolates 2 delta patterns | B4-51, B6-27 | A5-27 |  |  |  |  |  |  |  |  |  |  |  |  |  |  |  |  |  |
|  | XIX | 5 isolates 5 delta patterns | A5-34 | A5-37 | A6-8 | A7-46 | B4-23 |  |  |  |  |  |  |  |  |  |  |  |  |  |  |
|  | XX | 3 isolates 2 delta patterns | A5-35, A6-11 | A6-20 |  |  |  |  |  |  |  |  |  |  |  |  |  |  |  |  |  |
|  | XXI | 3 isolates 2 delta patterns | B4-39, B6-15 | A5-38 |  |  |  |  |  |  |  |  |  |  |  |  |  |  |  |  |  |
|  | XXVI | 6 isolates 4 delta patterns | A5-49, F1-9 | D3-9 | B6-37, B6-42 | B6-4 |  |  |  |  |  |  |  |  |  |  |  |  |  |  |  |
|  | XXXI | 3 isolates1 delta pattern | A6-13, A6-22, A7-23 |  |  |  |  |  |  |  |  |  |  |  |  |  |  |  |  |  |  |
|  | XXXII | 2 isolates 1 delta pattern | A6-17, A6-18 |  |  |  |  |  |  |  |  |  |  |  |  |  |  |  |  |  |  |
|  | XXXIV | 2 isolates 2 delta patterns | A6-23 | B6-30 |  |  |  |  |  |  |  |  |  |  |  |  |  |  |  |  |  |
|  | XLIII | 4 isolates 2 delta patterns | A7-3, A7-7, A7-20 | A7-41 |  |  |  |  |  |  |  |  |  |  |  |  |  |  |  |  |  |
|  | XLIV | 2 isolates 2 delta patterns | A7-4 | B6-34 |  |  |  |  |  |  |  |  |  |  |  |  |  |  |  |  |  |
|  | XLVII | 2 isolates 2 delta patterns | A7-19 | A7-36 |  |  |  |  |  |  |  |  |  |  |  |  |  |  |  |  |  |
|  | IL | 2 isolates 2 delta patterns | A7-38 | G1-6 |  |  |  |  |  |  |  |  |  |  |  |  |  |  |  |  |  |
|  | LIV | 4 isolates 1 delta pattern | B4-9, B4-27, B4-31, F1-45 |  |  |  |  |  |  |  |  |  |  |  |  |  |  |  |  |  |  |
|  | LV | 2 isolates 1 delta pattern | B4-11, B4-36 |  |  |  |  |  |  |  |  |  |  |  |  |  |  |  |  |  |  |
|  | LXII | 2 isolates 1 delta pattern | B4-42, B6-23 |  |  |  |  |  |  |  |  |  |  |  |  |  |  |  |  |  |  |
|  | LXXIV | 34 isolates 6 delta patterns | D2-4, D2-5, D2-6, D2-11, D2-13, D2-14, D2-16, D2-17, D2-20, D2-22, D2-23, D2-24, D2-26, D2-27, D2-28, D2-29, D2-30, D2-33, D2-34, D2-39, D2-41 | D2-46, D2-47, D2-50, D2-52, D3-3, D3-12 | D2-32, D3-34 | D2-48 | D2-51 | D2-35, D2-37, D2-44 |  |  |  |  |  |  |  |  |  |  |  |  |  |
|  | LXXVI | 4 isolates 4 delta patterns | D3-24 | D3-6 | D2-8 | D2-38 |  |  |  |  |  |  |  |  |  |  |  |  |  |  |  |
|  | LXXIX | 5 isolates 4 delta patterns | D3-37, D3-51 | D2-12 | D3-39 | D3-17 |  |  |  |  |  |  |  |  |  |  |  |  |  |  |  |
|  | LXXXI | 2 isolates 1 delta pattern | D2-18, D2-42 |  |  |  |  |  |  |  |  |  |  |  |  |  |  |  |  |  |  |
|  | LXXXII | 4 isolates 2 delta patterns | D2-19, D2-43, D3-47 | D2-31 |  |  |  |  |  |  |  |  |  |  |  |  |  |  |  |  |  |
|  | LXXXIII | 2 isolates 2 delta patterns | D2-21 | D2-45 |  |  |  |  |  |  |  |  |  |  |  |  |  |  |  |  |  |
|  | LXXXIV | 2 isolates 2 delta patterns | D2-25 | D2-49 |  |  |  |  |  |  |  |  |  |  |  |  |  |  |  |  |  |
|  | LXXXVII | 3 isolates 1 delta pattern | E1-7, E1-28, E1-36 |  |  |  |  |  |  |  |  |  |  |  |  |  |  |  |  |  |  |
|  | XCV | 3 isolates 2 delta patterns | E1-40, G1-22 | E1-51 |  |  |  |  |  |  |  |  |  |  |  |  |  |  |  |  |  |
|  | CVI | 6 isolates 5 delta patterns | F1-50, F1-51 | G1-38 | G1-4 | G1-23 | G1-48 |  |  |  |  |  |  |  |  |  |  |  |  |  |  |
|  | CIX | 2 isolates 1 delta pattern | G1-7, G1-15 |  |  |  |  |  |  |  |  |  |  |  |  |  |  |  |  |  |  |
|  | CX | 2 isolates 2 delta patterns | G1-9 | G1-10 |  |  |  |  |  |  |  |  |  |  |  |  |  |  |  |  |  |
|  | CXII | 2 isolates2 delta patterns | G1-12 | G1-13 |  |  |  |  |  |  |  |  |  |  |  |  |  |  |  |  |  |
|  | CXIII | 2 isolates 1 delta pattern | G1-14, G1-36 |  |  |  |  |  |  |  |  |  |  |  |  |  |  |  |  |  |  |
|  | CXIV | 2 isolates 1 delta pattern | G1-16, G1-43 |  |  |  |  |  |  |  |  |  |  |  |  |  |  |  |  |  |  |
|  | CXV | 3 isolates 1 delta pattern | G1-18, G1-35, G1-39 |  |  |  |  |  |  |  |  |  |  |  |  |  |  |  |  |  |  |
|  | CXVIII | 2 isolates 2 delta patterns | G1-27 | G1-46 |  |  |  |  |  |  |  |  |  |  |  |  |  |  |  |  |  |
|  | CXXII | 12 isolates 5 delta patterns | D3-1, D3-18, D3-36, D3-38 | D3-11, D3-14, D3-15 | D3-27 | D3-20, D3-31 | D3-5, D3-10 |  |  |  |  |  |  |  |  |  |  |  |  |  |  |
|  | CXXVII | 5 isolates 1 delta pattern | D3-30, D3-44, D3-49, D3-50, D3-52 |  |  |  |  |  |  |  |  |  |  |  |  |  |  |  |  |  |  |
