# Supplementary material for: Biodiversity of Indigenous Saccharomyces Populations from Old Wineries of South-Eastern Sicily (Italy): Preservation and Economic Potential
Source: PLoS One. 2012 Feb 29;7(2):e30428. doi: 10.1371/journal.pone.0030428 (PMC3290603; doi:10.1371/journal.pone.0030428)
Supplement: Table S5 — Acid content (g/l) at the end of malolactic fermentation. (DOC) [file pone.0030428.s009.doc]

**Supplementary table 5**

|  | Nero d’Avola B2-25 | Nero d’Avola B2-48 | Nero d’Avola ICV-D254 | Nero d’Avola QD145 | Frappato B2-25 | Frappato B2-48 | Frappato ICV-D254 | Frappato QD254 |
| --- | --- | --- | --- | --- | --- | --- | --- | --- |
| Malic Acid | 0.05 | 0.04 | 0.02 | 0.04 | 0.06 | 0.08 | 0.06 | 0.08 |
| Lactic acid | 1.21 | 1.30 | 0.94 | 1.05 | 1.25 | 1.22 | 0.91 | 1.05 |
| Total Acid | 4.20 | 4.50 | 4.30 | 4.20 | 4.70 | 4.90 | 4.50 | 4.80 |
