# Supplementary material for: Biodiversity of Indigenous Saccharomyces Populations from Old Wineries of South-Eastern Sicily (Italy): Preservation and Economic Potential
Source: PLoS One. 2012 Feb 29;7(2):e30428. doi: 10.1371/journal.pone.0030428 (PMC3290603; doi:10.1371/journal.pone.0030428)
Supplement: Table S6 — Average composition of single volatile compounds in Nero d'Avola (NdA) and Frappato wines. (DOCX) [file pone.0030428.s010.docx]

**Table S6**

Average composition of single volatile compounds in Nero d’Avola (NdA) and Frappato wines

|  | **NdA B2-25** | **NdA B2-48** | **NdA ICV-D 254** | **NdAQD-145** | **Frappato B2-25** | **Frappato B2-48** | **Frappato ICV-D254** | **Frappato QD-145** | **Odour threshold** | **Odour Characteristics** | |
| --- | --- | --- | --- | --- | --- | --- | --- | --- | --- | --- | --- |
| **Alcohols** (ppm) |  |  |  |  |  |  |  |  |  |  |  |
| Isoamyl alcohol | 130.46 | 104.19 | 107.39 | 99.29 | 103.97 | 94.78 | 71.55 | 104.12 | 30^[35]^ | Fruity, wine like |  |
| 1-hexanol | 2.20 | 2.57 | 3.14 | 2.74 | 0.49 | 0.58 | 0.42 | 0.53 | 0.5^[36]^ | Resin. flower. green |  |
| β-phenyl ethyl alcohol | 58.62 | 33.68 | 21.46 | 31.89 | 18.26 | 28.42 | 13.65 | 32.36 | 10^[36]^ | Floral. rose |  |
| *All* | *191.28* | *140.44* | *131.99* | *133.92* | *122.72* | *123.78* | *85.62* | *137.01* |  |  |  |
| **Acids** (ppm) |  |  |  |  |  |  |  |  |  |  |  |
| Octanoic acid | 0.42 | 0.26 | 0.28 | 0.22 | 0.09 | 0.09 | 0.09 | 0.09 | 7^[36]^ | Sweat. cheese |  |
| **Esters** (ppb) |  |  |  |  |  |  |  |  |  |  |  |
| Ethyl butanoate | 0.90 | 1.08 | 1.45 | 1.04 | 2.03 | 1.54 | 1.17 | 1.31 | 0.13^[36]^ | Fruity. strawberry. hazelnut |  |
| Ethyl 2-methyl butanoate | 0.40 | 0.40 | 1.11 | tr | 0.85 | 0.39 | 0.57 | 0.56 | 0.1^[36]^ | Anise. sweet. strawberry |  |
| Ethyl 3-methyl butanoate | 0.78 | 0.56 | 0.97 | 1.23 | 0.93 | 0.56 | 0.42 | 0.51 | 0.1^[36]^ | Fruity |  |
| Isoamyl acetate | 34.77 | 22.94 | 39.31 | 39.31 | 45.55 | 26.25 | 36.12 | 44.38 | 30^[35]^ | Banana |  |
| Ethyl hexanoate | 61.94 | 69.38 | 71.32 | 66.08 | 73.17 | 78.23 | 41.61 | 57.26 | 14^[35]^ | Anice. fruity. liquerice |  |
| Hexyl acetate |  |  |  |  | 2.79 | 2.70 | 0.05 | 0.05 | 2^[36]^ | Fruity |  |
| Methyl octanoate |  |  |  |  | 0.38 | 0.05 | 1.93 | 5.39 | 4^[36]^ | Orange |  |
| Ethyl heptanoate | 0.69 | 0.72 | 0.84 | 0.54 | 1.21 | 0.86 | 1.28 | 1.79 | 2^[36]^ | Fruity apricot |  |
| Ethyl octanoate | 302.26 | 297.46 | 278.08 | 227.88 | 257.60 | 276.82 | 231.08 | 246.33 | 8-12^[36]^ | Fruity fat |  |
| Isoamyl hexanoate | 2.83 | 2.30 | 1.43 | 1.92 | 2.10 | 2.14 | 1.51 | 2.02 | 2^[39]^ | Fruity. banana. apple |  |
| Methyl decanoate | 0.32 | 0.44 | tr | tr | 0.05 | 0.62 | 0.57 | 0.05 | 6^[36]^ | Wine |  |
| Ethyl decanoate | 120.41 | 102.53 | 94.73 | 75.03 | 51.77 | 91.95 | 109.23 | 91.38 | 8-12^[36]^ | Grape |  |
| Isoamyl octanoate | 4.40 | 3.16 | 2.07 | 2.31 | 2.67 | 2.52 | 2.82 | 3.10 | 1000^[39]^ | Fruity. peach |  |
| Diethyl succinate | 8.11 | 15.40 | 16.25 | 16.57 | 2.80 | 5.32 | 4.07 | 5.08 | 200^[36]^ | Wine fruity |  |
| Ethyl (Z)-9-decenoate | 3.94 | 2.39 | tr | 1.56 | 3.07 | 2.84 | 2.69 | 3.07 |  | Fruity |  |
| Methyl dodecanoate | 0.46 | 0.46 | 2.86 | 0.25 | 0.62 | 2.04 | 2.14 | 1.61 |  |  |  |
| Ethyl dodecanoate | 3.23 | 3.01 | 3.06 | 2.00 | 1.26 | 2.95 | 4.23 | 3.48 | >800^[39]^ | Sweet. floreal. fruity. cream |  |
| β-phenyl ethyl acetate | 6.45 | 4.86 | 5.95 | 5.49 | 0.49 | 0.61 | 0.68 | 0.72 | 250^[38]^ | Fruity. sweet |  |
| *All* | *551.89* | *527.09* | *519.43* | *441.21* | *449.34* | *498.39* | *442.17* | *468.09* |  |  |  |
| **Terpenes and C13 norisoprenoids** (ppm) |  |  |  |  |  |  |  |  |  |  |  |
| Limonene | 0.003 | 0.004 | 0.005 | 0.003 | tr | tr | tr | tr | 0.015^[37]^ | Lemon. citrus |  |
| Terpinolene | tr | 0.001 | tr | tr | tr | tr | tr | tr |  | Fresh. pine |  |
| Linalyl acetate | 0.001 | 0.001 | 0.001 | 0.001 |  |  |  |  |  |  |  |
| Linalool | 0.032 | 0.024 | 0.024 | 0.024 | 0.039 | 0.043 | 0.061 | 0.044 | 0.025^[35]^ | Floral. lavender |  |
| α-ionone | 0.039 | 0.028 | 0.038 | 0.041 | 0.054 | 0.065 | 0.065 | 0.051 | 0.0026^[37]^ | Flower. violet |  |
| β-ionone | 0.024 | 0.019 | 0.022 | 0.024 |  |  |  |  | 7^[42]^ | Flower. violet |  |
| α-terpineol | tr | tr | tr | tr | 0.016 | 0.017 | 0.022 | 0.019 | 0.25^[37]^ | Lily of the valley. lilac |  |
| α-muurolene | tr | tr | tr | tr | tr | tr | tr | tr |  | Wood |  |
| (E)-nerolidol | tr | tr | tr | tr |  |  |  |  | 0.25^[41]^ | Floral. wood |  |
| β-pinene |  |  |  |  | tr | tr | tr | tr |  | Pine . resin. turpentine |  |
| α.-terpinene |  |  |  |  | tr | tr | tr | tr |  | Lemon |  |
| γ-terpinene |  |  |  |  | 0.016 | tr | 0.012 | 0.010 |  | Gasoline. turpentine |  |
| o-cimene |  |  |  |  | 0.016 | 0.014 | 0.015 | 0.017 |  | Herb |  |
| (E)-rose oxide |  |  |  |  | 0.031 | 0.032 | 0.034 | 0.037 | 0.0002^[35]^ | Flower. rose |  |
| (Z)-rose oxide |  |  |  |  | tr | 0.018 | tr | 0.004 |  | Flower. rose |  |
| (E)-linalolo oxide |  |  |  |  | 0.028 | 0.022 | 0.028 | 0.034 | 0.5^[40]^ | Floral. rose. wood |  |
| Geranyl ethyl ether |  |  |  |  | 0.013 | 0.018 | 0.017 | 0.019 |  |  |  |
| *All* | *0.099* | *0.078* | *0.090* | *0.095* | *0.213* | *0.229* | *0.255* | *0.234* |  |  |  |

tr< 1ppb
